# Genome scan identifies flowering-independent effects of barley HsDry2.2 locus on yield traits under water deficit

**DOI:** 10.1093/jxb/ery016

**Published:** 2018-01-08

**Authors:** Lianne Merchuk-Ovnat, Roi Silberman, Efrat Laiba, Andreas Maurer, Klaus Pillen, Adi Faigenboim, Eyal Fridman

**Affiliations:** 1 Institute of Plant Sciences, Agricultural Research Organization (ARO), The Volcani Center, Bet Dagan, Israel; 2 The Smith Institute of Plant Sciences and Genetics in Agriculture, The Hebrew University of Jerusalem, Rehovot Israel; 3 Institute of Agricultural and Nutritional Sciences, Martin-Luther University Halle-Wittenberg, Halle (Saale), Germany

**Keywords:** Adaptation, drought, flowering, genotype by environment interactions, grain yield, wild barley

## Abstract

Increasing crop productivity under conditions of climate change requires the identification, selection, and utilization of novel alleles for breeding. In this study, we analysed the genotype and field phenotype of the barley HEB-25 multi-parent mapping population under well-watered and water-limited environments for two years. A genome-wide association study (GWAS) for genotype × environment interactions was performed for 10 traits including flowering time (heading time, HEA) and plant grain yield (PGY). Comparison of the GWAS for traits *per se* (i.e. regardless of the environment) with a study for quantitative trait loci (QTLs) × environment interactions (Q×E), indicates the prevalence of Q×E mostly for reproductive traits. One Q×E locus on chromosome 2, *Hordeum spontaneum* Dry2.2 (HsDry2.2), showed a positive and conditional effect on PGY and grain number (GN). The wild allele significantly reduced HEA; however, this earliness was not conditioned by water deficit. Furthermore, BC_2_F_1_ lines segregating for the HsDry2.2 locus showed that the wild allele conferred an advantage over the cultivated allele in PGY, GN, and harvest index, as well as modified shoot morphology, a longer grain-filling period, and reduced senescence (only under drought). This suggests the presence of an adaptation mechanism against water deficit rather than an escape mechanism. The study highlights the value of evaluating wild relatives in search of novel alleles and provides clues to resilience mechanisms underlying crop adaptations to abiotic stress.

## Teaser

The wild barley HsDry2.2 allele confers a reproductive advantage that is independent of flowering time under drought conditions and is manifested through modifications in shoot morphology, reduced senescence, and longer grain filling.

## Introduction

Barley (*Hordeum vulgare* ssp. *vulgare* L.) is ranked the fourth most-produced cereal worldwide, providing fodder, human food, and substrate for malting (Druka *et al.*, 2011). Changing climate and increasing aridity poses a threat to future global food security (Wheeler and von Braun, 2013), with drought stress, the major factor limiting crop yield (Boyer, 1982; Araus *et al.*, 2008), expected to further increase (Wheeler and von Braun, 2013). It is therefore necessary to invest efforts into breeding-based improvements of crop resilience to abiotic stresses and into enhancement of plant yield robustness across a range of environments (Cattivelli *et al.*, 2008; Tester and Langridge, 2010). Domestication of barley, which took place approximately 10500 years ago (Mascher *et al.*, 2016), and its subsequent genetic selection has led to genetic erosion (Tanksley and McCouch, 1997). Wild relatives of crops, which harbor most of the pre-domestication gene pool, may serve as valuable sources in attempts to formulate new genetic variation to improve drought resistance in modern varieties (Ellis *et al.*, 2000; Zamir, 2001).

Traits enabling drought tolerance may involve adaptive phenological and cellular processes that are responsive to water stress. These include ‘escape mechanisms’, via extremely early flowering that occurs at the expense of a shortened growth cycle, and ‘drought avoidance’ in which active accumulation of solutes (reducing osmotic potential to more negative values) retains water in the cells (i.e. osmotic adjustment) and thus sustains metabolic activity (Blum, 2005). Plant survival requires conserved water status and that is usually accompanied by inhibition of growth. However, yield stability requires the maintenance of, or an increase in, sink activity in the reproductive structures, which contributes to the transport of assimilates from the source leaves and to delayed stress-induced leaf senescence (Albacete *et al.*, 2014). A considerable number of genes have been suggested to be involved in drought tolerance, and many have been discovered using differential genomics methods (Rollins *et al.*, 2013; Bedada *et al.*, 2014). Such methods ignore the whole-plant perspective, which is key to understanding the subtle sink–source relationship and its optimal maintenance for yield stability. Genome scans in search of reproducible interactions between quantitative trait loci (QTLs) and environment loci (Q×E) under field conditions (which take into account possible pleiotropic effects, or lack thereof) provide a promising entry point for deciphering the major drivers of pathways that confer drought tolerance in the field.

Advanced backcross QTL analysis (AB-QTL) enables the introduction of beneficial alleles into the modern gene pool by crossing a wild donor accession with a modern elite cultivar (Fulton *et al.*, 2000; Pillen *et al.*, 2003; Talamè *et al.*, 2004; Von Korff *et al.* 2005, 2006, 2008, 2010), followed by a number of selfings. In addition, recombinant inbred line (RIL) populations, derived from crossings of a wild donor with a cultivar (or between two distinct domesticated parental lines), have been used to map QTLs for grain yield and yield components under reduced moisture (Teulat *et al.*, 2003; Kirigwi *et al.*, 2007). As noted by Comadran *et al.* (2011), in contrast to the limited allelic diversity in such bi-parental-based genetic structures, recently developed multi-parental populations, which combine linkage and genome-wide association (GWA) approaches, offer a much wider genetic variance. GWA studies (GWAS) have seldom been used to detect Q×E interactions, mainly due to a lack of statistical power resulting from the frequent occurrence of rare alleles that are difficult to detect in a GWAS, but which appear to contribute to strong genotype × environment interactions (Thomas, 2010). Notably, in most of these studies, the genetic model undertaken compares the GWAS results under one environment versus another in order to identify environment-specific QTLs. Very few studies have considered marker × environment interactions in their genetic model (Francia *et al.*, 2011), and hence they have precluded testing of the same single-nucleotide polymorphisms (SNPs) across all environments and reduced the ability to discover novel alleles or genes that synergistically contribute to environmental adaptation and plasticity (El-Soda *et al.*, 2014). From a breeding point of view, constitutive QTLs are the main targets for breeding programs, as they show a consistent effect across environments (Comadran *et al.*, 2011; Korte *et al.*, 2012). However, if the goal is to understand the mechanism underlying genotype × environment interactions, then conditioned QTLs are imperative targets for follow-up studies.

The barley nested association mapping (NAM) population termed ‘Halle Exotic Barley 25’ (HEB-25) originated from interspecific crosses between the spring barley elite cultivar Barke (*Hordeum vulgare* ssp. *vulgare*) and 25 highly divergent exotic wild barley genotypes (*H. vulgare* ssp. *spontaneum*). The population has been used to study the genetic architecture of flowering (Maurer *et al.*, 2015) and grain weight (Maurer *et al.*, 2016). The NAM approach (Buckler *et al.*, 2009) was originally designed to overcome GWAS limitations, such as the incidence of false positives resulting from population structure. In the current study, NAM was harnessed to evaluate marker × environment interactions. Each of the 1420 HEB-25 BC_1_S_3_ lines and their corresponding parents were genotyped using the Infinium iSelect 9K chip, which consists of 7864 SNPs (Comadran *et al.*, 2012). Previously, a combined linkage and GWAS analysis of HEB-25 identified eight major QTLs that control flowering time, potentially explaining the QTL effect. Most co-located with major flowering genes, including *Ppd-H1*, *HvCEN* (both on chromosome 2H), and *Vrn-H1/H2/H3* (on chromosomes 5H, 4H, and 7H, respectively). The strongest exotic haplotype identified accelerated flowering time by 11 d, as compared to Barke (Maurer *et al.*, 2015). Similarly, Maurer *et al.* (2016) reported that grain weight was increased by 4.5 g and flowering time was reduced by 9.3 d after substituting Barke elite QTL alleles for exotic QTL alleles at the semi-dwarf locus *denso/sdw1* (3H) and the *Ppd-H1* loci, respectively. In a more recent use of this genetic resource, the plants were placed under two regimes of salinity and GWAs of the two treatments were compared (Saade *et al.* 2016). While marker × environment interactions were not reported, constitutive QTLs under both environments, which, from a breeding point of view are of high value, were identified.

In the current study, the BC_1_S_3_ HEB-25 families were used in a GWAS of the genetic architecture of drought response in relation to plant grain yield and related traits. A mixed linear model was used to test the Q×E interactions for the traits in order to identify specific loci that contribute to plant adaptation via dependent or independent effects on phenology and morphology. The analysis showed no interactions between flowering time loci, and identified several significant interactive QTLs that affect plant grain number and yield in a manner dependent on water deficiency. In addition, a pot experiment was carried out using an advanced backcross population segregating for the HsDry2.2 locus, which was found to improve yield when donated by the wild parent at this locus. This experiment was designed to evaluate the effect of early and late water limitation by measuring plant productivity, phenology, canopy structure, and leaf dimensions. This study highlights the power of integrating semi-controlled field experimental systems and interspecific multi-parental populations in pursuit of agricultural traits, which together may shed light on hitherto unknown mechanisms underlying crop adaptation.

## Materials and methods

### Plant material

#### Field trials

The NAM population HEB-25, which was developed by Maurer *et al.* (2015), consisted of 1420 BC_1_S_4_ lines belonging to 25 interspecific crosses between the elite cultivar Barke and wild barley accessions. All 1420 BC_1_S_3_ lines (one generation earlier) and their corresponding parents were genotyped using the barley Infinium iSelect 9K chip (Maurer *et al.*, 2015), consisting of 7864 SNPs (Comadran *et al.*, 2012). Inclusion of SNPs that were polymorphic in at least one HEB family and that met the pre-defined quality criteria [<10% missing, and not in complete linkage disequilibrium to another SNP in the set] resulted with 5709 informative loci.

#### Pot experiment

BC_2_F_1_ seeds (HEB-04-96) segregating for the wild donor allele in the HsDry2.2 locus were genotyped for the peak marker BOPA2_12_30265 using high-resolution melting analysis. This marker was previously found to show strong divergent selection in winter versus spring barleys according to Comadran *et al.* (2012). SYTO™ 9 Green Fluorescent Nucleic Acid Stain (ThermoFischer, 0.6 µl per 20 µl reaction) was used for PCR together with Taq ready mix (HyLabs), with the primers listed in Supplementary Table S1. Melting analysis was conducted using a RotorGene 6000 real-time PCR machine and software and validated by sequencing (Supplementary Fig. S1A, B). The Barke cultivar, which sets the genetic background, was also included and genotyped as a control. The different genotypes were grouped as carriers for the wild (Hs/Hs and Hv/Hs; *N*=22 and *N*=7, respectively) or cultivated (Hv/Hv; *N*=26) allele for statistical analyses.

### Field-trial growth conditions

The HEB-25 lines were evaluated for their drought responses under well-watered (WW) and water-limited (WL) conditions during the winters of 2014–15 (BC_1_S_3:4_) and 2015–16 (BC_1_S_3:5_). During 2014–15, the entire population was phenotyped in the field under both conditions, and in 2015–16 1320 lines were included in the experiment. Seeds from each line were sown in germination trays and at the 3-leaf stage a total of 16 plants were transplanted into troughs measuring 0.4 × 0.3 m (Mapal Horticulture Trough System, Merom Golan, Israel), with each trough containing eight plants. The soil in the troughs was composed of two layers of volcanic soil (4–20 type of rough soil topped by a finer Odem193 type; Toof Merom Golan, Merom Golan, Israel). The two troughs for each line were kept together in pairs and watering was regulated throughout the growth of the plants (see Results). The pairs of troughs for each line were placed in a completely randomized design within an insect-proof net house, roofed by polyethylene, at the experimental farm of The Hebrew University of Jerusalem in Rehovot, Israel (E34°47′, N31°54′; 54 m above sea level). The unique arrangement of the net house enabled approximately 3000 experimental units of eight plants to be accommodated (Supplementary Fig. S2A), with the capacity to have different irrigation between adjacent troughs. Three cultivated barley lines (Apex, Barke, and Bowman) served as controls for the effects of water deficit. To mimic the natural pattern of rainfall in the east Mediterranean region, water was applied during the winter months starting from planting (14 December In 2014 and 23 November In 2015) and ending in early spring (143 and 145 d after planting in 2014–15 and 2015–16, respectively; Supplementary Fig. S2B). In order to ensure adequate drought stress, irrigation was adjusted in accordance with the stomatal conductance determined for eight test plants per drought treatment during the growing season (at 57, 98, 110, and 127 d from planting in 2014–15, and at 70, 84, 119, and 154 d from planting in 2015–16), measured using a porometer (Decagon SC-1; Supplementary Fig. S2C). This ensured differences of ~30% between the WL and the WW plants, with maximal conductance of 350 and 250 mmol m^−2^ s^−1^ in the WL and WW treatments, respectively. The total seasonal water application consisted of 25 m^3^ and 34 m^3^ for the WW treatment, and 13 m^3^ and 26 m^3^ for the WL treatment, in 2014–15 and 2015–16, respectively. NPK fertilizer (Shefer 538+Microelements, Deshen Gat, Qiryat Gat) was applied via irrigation in 2014–15 (8.1 l and 6.5 l for the WW and the WL treatments, respectively) as well as in 2015–16 (15.4 l for both treatments).

### Pot experiment irrigation management

Seeds were placed in moist germination paper for a week in a dark cold room (4 °C), followed by 3 d of acclimation at room temperature (22 °C), and then planted into small plastic pots (60 g soil and 300 g water, 100% field capacity) in a standard commercial soil mix (Green 90, Even Ari, Israel; Supplementary Fig. S2D). At this time the early drought treatment was initiated. At 28 d after planting (DAP), plants were transplanted into medium pots (190 g soil and 1170 g water). Finally, at 52 DAP plants were transplanted into large pots (460 g soil and 2500 g water), with initiation of the late drought treatment at 13 d before booting (Supplementary Fig. S2E). Pots were weighed manually before and after irrigation, keeping the well-watered (WW) pots between ~60–90% of field capacity and the water-limited pots (WL) at ~40–60%. Temperatures were monitored using a data logger (Hobo Onset, Bourne, MA, USA).

### Phenotypic measurements

#### Field trials

Heading time (HEA), defined as the time between sowing to the time at which the first spikes of 50% of the plants in a plot have first awns visible (BBCH-scale 49), was recorded based on daily inspections. Days from sowing to stage BBCH 87 (hard dough; grain content solid; fingernail impression held) was recorded as maturity (MAT). At maturity, mean plant height (HEI) per plot was measured from the soil surface to the base of the three first spikes.

At full grain maturity and after plants were fully dried, all above-ground biomass was harvested and weighed to determine total dry matter (TDM). In particular, all the free-threshing material (approximately one quarter of the genotypes) was caged with netting bags between stages BBCH49 and BBCH87 to avoid loss of spikes. Spikes were then threshed and the grains were weighed to determine plant grain yield (PGY). Finally, grains were counted to estimate grain number (GN) per 8-plant trough and average grain weight (GW). Harvest index (HI) was calculated as the ratio between PGY and TDM. Vegetative dry weight (VDW) was calculated by subtracting PGY from TDM. The grain-filling period (GFP) was calculated by subtracting HEA from MAT. Trait values were adjusted based on the ratio between population mean values in the two years, with each trait value being multiplied a factor equal to 1/(mean of trait in individual experiment/mean of trait in both years). The adjusted means of the HEB family traits were then averaged across the two years and were used in the GWAS (see below).

#### Pot experiment

Heading time, defined as the date at which the awns of the first three spikes were first visible (spikes were tagged), was recorded daily and used to score HEA. MAT was recorded when the three first (tagged) spikes had dried, which was followed by the rest of the plant drying out. The GFP was calculated by subtracting HEA from MAT. All above-ground biomass per plant was harvested at full grain maturity; fertile spikes were counted to assess the number of spikes per plant and were separated from the vegetative organs (stems and leaves), both were oven-dried (80 °C or 38 °C for 48 h for vegetative organs and spikes, respectively) and weighed to determine spike dry matter and TDM. Spikes were threshed and the total grain weight was determined. PGY, HI, and GN per plant were determined. The mean GW for the tagged spikes was recorded, and the mean GW of the other spikes was determined separately. At 64 DAP, the flag leaf (FL) sheath and the blade length and width of the preceding leaf (termed here as ‘–1FL’) were measured. Stem diameter (adjacent to the spike) was measured after harvest. For analysis of leaf senescence, we used two sets of photos taken with a Canon EOS1200 camera at 66 and 78 DAP against a background of a standard white sheet (80 × 120 cm). Custom-made software (unpublished data) was used to calculate the ratio of yellow/brown areas to the total leaf area. Shades covering the range of green and yellow/brown were selected by sampling from several images, and were then used for all analyses. For each photo, the green and the yellow/brown areas were calculated in percentages, using the YCbCr method (pixels ‘close’ to one of the specified shades were considered part of the measured object, while pixels further away were considered background), with same tolerance level for all analyses, while taking into account the white background for standardization. Then, the percentage of yellow/brown out of the total leaf area was calculated. Senescence was calculated as the increase of yellow/brown to the total between 66 and 78 DAP for each plant.

### Field trials genome-wide association study

A genome-wide association study (GWAS) was conducted to identify trait variations, *per se* (i.e. regardless of environment), under WW and WL conditions, and to assess SNP interactions with the environment (conditional effects) using a mixed linear model (MLM) as implemented in the software package TASSEL 5.0 (Bradbury *et al.*, 2007). The marker–trait association was conducted with the adjusted means of the HEB lines, as detailed above (see Phenotypic measurements). The genetic model used was as follows:

where µ denotes the population mean for the trait, *F*_*i*_ is the family effect (*i*=1..25), *G*_*j*_ denotes the marker effect (including heterozygous, i.e. *j*=1..3), *K* represents the relative kinship matrix, and ε_*ij*_ denotes the error.

A genome scan for SNP × environment (Q×E) interactions was conducted with the GWAF package in the R software (http://cran. r-project.org/web/packages/GWAF/). To test associations between each of the continuous traits and each SNP, we applied the linear mixed effects model (LME) implemented in lmekin.int.batch in the GWAF package based on the kinship computed by the kinship2 package (http://cran.r-project.org/web/packages/kinship/; version 1.6.4) with addition of the WW/WL conditions as follows:

where, *E*_*k*_ denotes the watering (environment) effect and *G*_*j*_ × *E*_k_ denotes the effect of the interaction between the marker and the environment. The in-house R script that utilizes the GWAF and kinship2 R-packages can be found in Supplementary File S1.

To test the robustness of the association for each SNP, the procedure was cross-validated 200 times on random sub-samples of the full dataset. Each subsample included 70% of the lines, randomly selected per HEB family. Markers that were significantly detected (*P*<0.05) in at least 30% of subsamples were accepted as putative QTLs. The designation of the QTL was based on linkage disequilibrium with the major SNP that showed the maximal significance level in a genomic interval (Maurer *et al.*, 2015).

### Pot experiment statistics

A factorial model was employed for the ANOVA (Supplementary Table S6) with drought treatment and allelic state as fixed effects. There were at least six replicates for each combination (three drought treatments × two allelic states). Each experimental unit consisted of a pot with one plant. The statistical package JMP version 12.0 (SAS Institute, Cary, NC, USA) was used for analyses.

### Allele mining in diverse barley HEB accessions

The plant material used for sequencing *HvCEN* in this study included representatives of cultivated and wild alleles from the HEB-04 and HEB-05 families and is described in Supplementary Fig. S7. Three primer pairs (Supplementary Table S1) flanked and included all ORFs, including two SNPs that distinguish the wild allele of HEB-04 from the cultivated one (Supplementary Fig. S7). All DNA amplifications were performed in a 25-μl reaction volume using standard conditions and were sequenced on an ABI Prism 3730xl sequencer using BigDye terminators at HyLab labs (Rehovot, Israel). Sequence alignments were generated with the Geneious software (version R10.2, http://www.geneious.com;Kearse *et al.*, 2012). GenBank accessions for *HvCEN* alleles from four HEB genotypes are: MG732902, HEB-04-02; MG732903, HEB-04-96; MG732904, HEB-05-140; MG732905, HEB-05-153.

## Results

### Whole-plant phenotypes of the HEB-25 multi-parent population under well-watered and water-limited conditions

The HEB-25 family was grown in troughs of eight plants during 2014–15 and 2015–2016 (hereafter 2015 and 2016). The experimental set-up under a rain-sheltered net house was designed to allow whole-plant phenotyping of plants together with controlled irrigation with measurable water deficit in the plants (Supplementary Fig. S2). Assessment of the effect of water-limited (WL) conditions on control plants that were distributed across the entire net house system clearly indicated mild, yet statistically significant differences in the stomatal conductance between well-watered (WW) and WL conditions, starting at least 70 d after sowing (no porometer measurements were made for younger plants) and onward (Supplementary Fig. S2C).

**Fig. 1.**
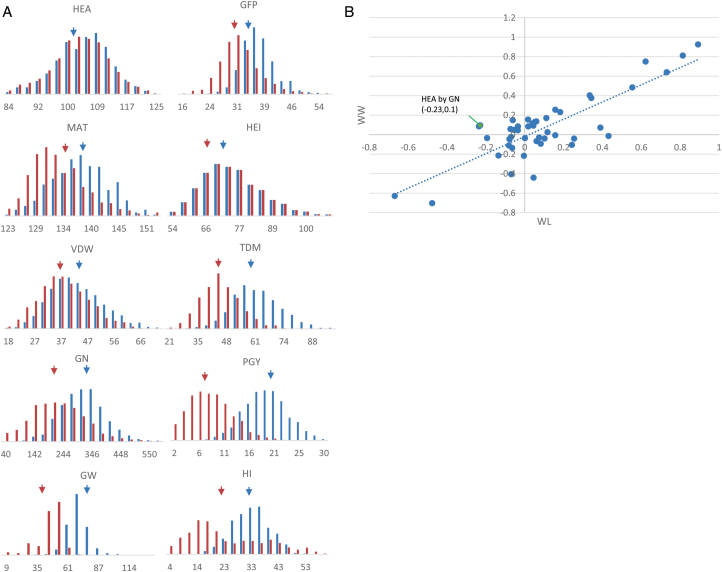
Distribution and correlation matrix of the measured traits under well-watered (WW) and water-limited (WL) conditions. (A) The distribution of the traits for the HEB-25 lines for WW (blue) and WL (orange) drought conditions (see detailed values in Supplementary Table S2). The blue and orange arrows indicate the mean values under WW and WL environments, respectively. (B) Scatter-plot of the pairwise correlations between the 10 traits under WL and WW. HEA, time to heading (days from sowing to anthesis); MAT, time to maturity (days from sowing to maturation); GFP, grain filling period (d); HEI, plant height (cm); VDW, vegetative dry matter per plant (g); TDM, total dry matter per plant (g); PGY, plant grain yield (g); GN, grain number per plant; GW, grain weight per plant (mg); HI, harvest index.

Overall, we observed wide variation for all traits across the HEB-25 population, and increases in the coefficient of variation under WL conditions, with means of 28.3 versus 16.8 for all traits under WL versus WW, respectively (Fig. 1A, Supplementary Table S2). WL conditions significantly reduced the means of all measured traits as compared to WW, with effects on heading time (HEA) being the mildest (–1.2%). On average, plant grain yield (PGY) and grain number (GN) were reduced by –58.3% and –31%, respectively. The effects of WL on vegetative growth were weaker, with a –13% reduction in both vegetative dry weight (VDW) and plant height (HEI).

Pair-wise correlation analysis between all traits found similar relationships under both treatments, with a few exceptions (Fig. 1B, Supplementary Table S3). One such exception was the relationship between HEA and GN: under WW conditions there was a positive and slight correlation between these traits (*r*=0.1, *P*<0.005), whereas under WL this correlation was negative (*r*=–0.23, *P*<0.0001).

### GWAS for Q×E interactions

**Fig. 2.**
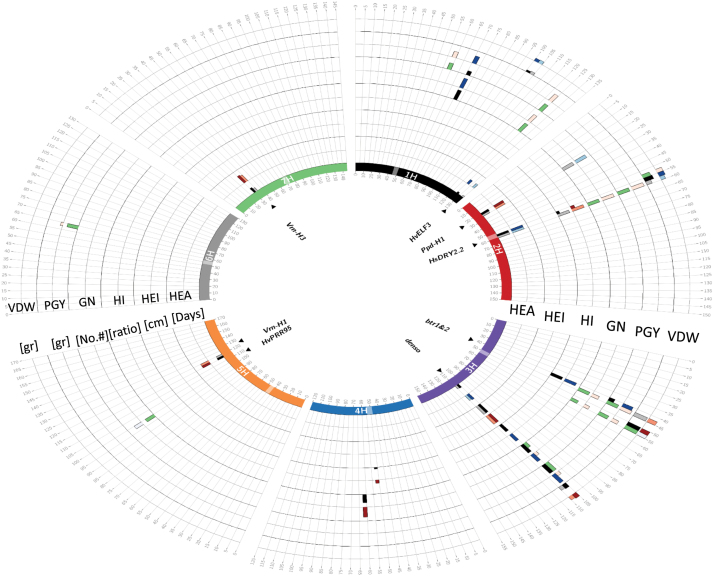
Circos plot depicting the GWAS results for traits *per se* (QTL) and interactions with environment (Q×E). Barley chromosomes in the plot are depicted in different colors in the inner-most circle and centromeres are indicated by the boxes radiating outwards. For each trait, the first (inner) track represents the QTL detection rate calculated across 200 repeated subsamples of 70% from the HEB-25 population in a 5-cM window, and the adjacent (outer) track represents the effect of this QTL. The maximum height of the effect bars is 10.03 d for HEA, 17.34cm for HEI, 24.3 for HI, 22.5 for GN, 22.44 g for PGY, and 23.04 gfor VDW. Window positions (in cM, as per Maurer *et al.* 2015) are ordered clockwise, per chromosome. In the inner track, QTLs appearing under WW and WL conditions are presented by black and gray bars, respectively. The QTLs showing significant Q×E interactions are represented by green bars. The effect of the QTL conferred by the wild allele relative to cultivated Barke is represented on the outer track, where blue and red bars indicate decreasing and increasing wild barley QTL effects, respectively, for each treatment. Genes potentially explaining the observed QTL effects are indicated inside the inner circle.

Initially, we performed a genome scan for all ten traits under both watering treatments, and we identified 69 loci with a significant contribution to trait variation (Fig. 2, Supplementary Table S4). The main loci for HEA, including *HvELF3*, *Ppd-H1*, *HvCEN*, *denso*, *Vrn-H1*, and *Vrn-H3*, were identified. In general, at loci associated with PGY and GN the wild alleles were associated with a reduction of the traits under WW conditions. Under this trait analysis, the mean reductions of the three loci significantly associated with GN or PGY variation were –3.4% and –9.2%, respectively, under WW conditions (Supplementary Table S4). Similar effects were observed under WL conditions for a locus linked with *Ppd-H1* on chromosome 2 (–19.7% effect with probability of association –Log*P*=7.9) for GN.

Next, the GWAS model was modified to identify QTL × environment interactions (Q×E; see Methods). No significant Q×E loci were identified for two of the phenological traits, HEA and MAT. For the vegetative traits, only two Q×E loci were identified in VDW and no significant loci were identified for plant height (Supplementary Table S5). In contrast, a larger number of interactive loci were associated with variations in PGY and GN (Fig. 2). In most of these loci the wild allele was characterized with a conditional beneficial effect on the trait, i.e. carriers of the wild allele were less affected by the water deficit across the two environments.

### HsDry2.2, a major locus with non-pleiotropic Q×E interactions

**Fig. 3.**
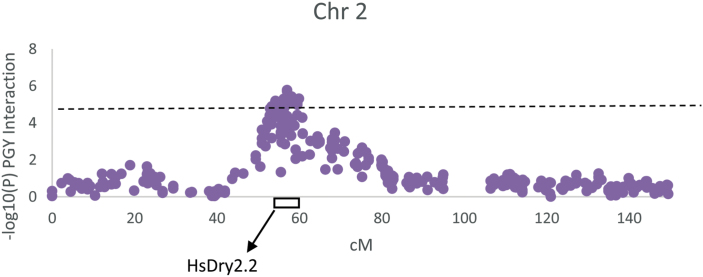
Manhattan plot of the genome-wide association for Q×E interactions for plant grain yield (PGY) in chromosome 2. The plot indicates the location of the HsDry2.2 locus on the long arm according to genetic mapping. The *y*-axis depicts the –log(*P*) value of the Q×E interaction. It is delimited by SCRI_RS_144592 and SCRI_RS_165574 (54.2–59.9 cM), peaking at 57cM.

**Fig. 4.**
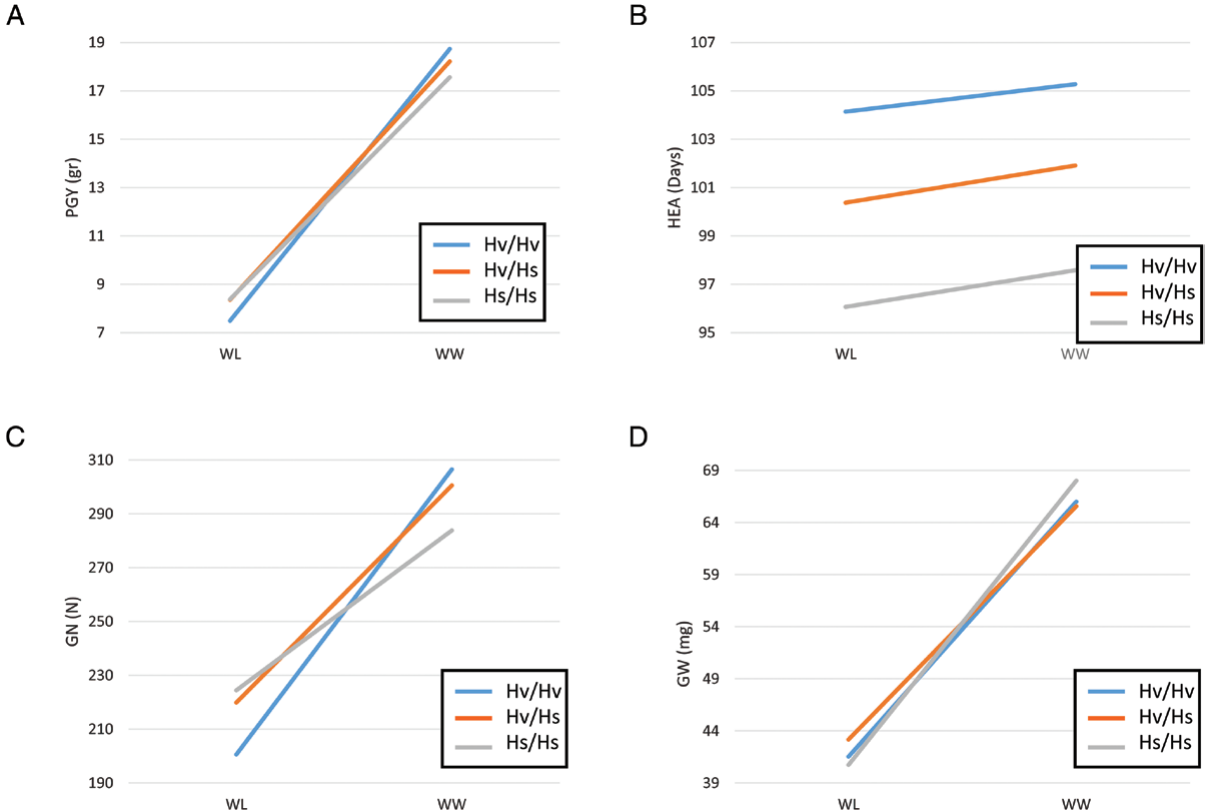
Least-square mean value comparisons (reaction norms) for the HsDry2.2 genotypes under well-watered (WW) versus water-limited (WL) conditions in the whole HEB-25 population based on the BOPA2_12_30265 marker. (A) Plant grain yield (PGY), (B) days from sowing to heading (HEA), (C) grain number (GN), and (D) grain weight (GW). The three genotypic groups of plants are homozygous for the Barke cultivated allele (Hv/Hv), homozygous for the wild allele (Hs/Hs), and heterozygous (Hv/Hs).

One major locus that appeared as a pleiotropic QTL that regulates many traits was located on the long arm of chromosome 2 and delimited by SCRI_RS_144592 and SCRI_RS_165574 (54.2–59.9 cM; Fig. 3). We named this locus *Hordeum spontaneum* Dry 2.2 (HsDry2.2). The peak of the Q×E interaction in this locus was identified at 57 cM. Plotting the mean values of PGY measured for the HEB-25 population under WW and WL conditions, using BOPA2_12_30265 as a representative marker, illustrates the conditioned effect of HsDry2.2 on the trait (Fig. 4A). This is in contrast to identical effects under both growth conditions of the wild allele on HEA and VDW (no interaction; Fig. 4B; Supplementary Fig. S3), i.e. a reduction of the days to heading by an average of 7.7 and 8.1 d in WW and WL, respectively. Conditioned effects on GN were more pronounced (Fig. 4C), but no significant effect on mean grain weight (GW; Fig. 4D) was associated with the locus.

Dissection of the family-specific effects of HsDry2.2 on GN suggested that the causal allele or alleles for this significant Q×E interaction (*P*<0.01) originated from at least three wild donors. Performing the genotype-to-phenotype analysis in the HEB-16 family showed that plants carrying the wild HID219 allele (Maurer *et al.* 2015) exhibited a significant reduction in GN between WW and WL conditions (334 versus 221 in WW and WL, respectively, i.e., a reduction of 34% under water deficit) in a similar manner to that of the cultivated-allele carriers (Supplementary Fig. S4A). In contrast, a much less pronounced reduction in plants carrying the wild allele was observed in the HEB-5 family (HID065 as donor), with a reduction of 7.3% from 272 to 252 when homozygous to the wild allele (Supplementary Fig. S4B). These differences translated to a family-specific positive conditioning effect of the wild barley allele under WL conditions.

### The effect of HsDry2.2 in consecutive BC_2_S_1_ population

Plants derived from one of the HEB lines that carry the HsDry2.2 *H. spontaneum* (HID062) allele were further analysed to validate effects on reproductive traits, as well on possible related traits. A pot experiment was carried out to evaluate the wild allele effects under optimal as well as early and late water limitation, i.e. starting at transplanting or at the stem elongation stage (52 DAP), respectively. The type of traits examined were plant productivity, phenology, canopy structure, and leaf senescence.

#### Overall plant performance

**Fig. 5.**
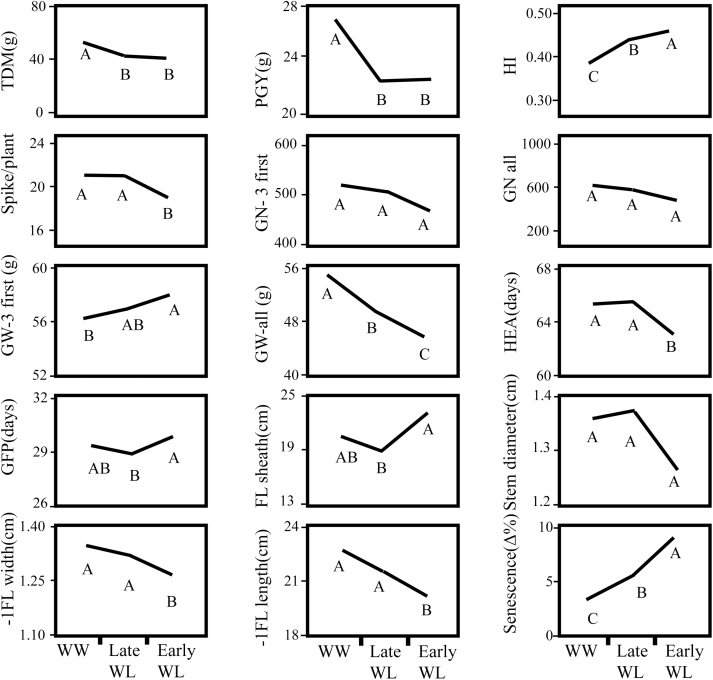
Effects of drought treatment on the different plant parameters: TDM, total dry matter per plant (g); PGY, plant grain yield (g); HI, harvest index; spikes per plant; GN-3 first, grain number of first three spikes; GN-all, grain number of all spikes; GW-3 first, grain weight of first three spikes (g); GW-all, grain weight of all spikes; HEA, days from planting to heading; GFP, grain filling period (d); FL, flag leaf sheath length (cm); stem diameter (cm); –1FL width, blade width of the leaf preceding the flag leaf (cm); -–FL length, blade length of the leaf preceding the flag leaf (cm); and senescence, change in % area of yellow-brown coloration on the leaf between 66–78 d after planting. Different letters indicate significant differences at P<0.05 (Student’s t-test).

For most of the measured variables the effect of the drought treatment was found significant (Fig. 5, Supplementary Table S6). TDM under the early and late WL treatments was reduced by 31% and 26%, respectively, as compared to the WW treatment. GY was reduced by 17% and 16% for the early and late WL as compared to the WW. HI increased under both WL treatments as compared to the WW (16% and 12% for early and late, respectively). For the leaf preceding the flag leaf (–1FL), there were reductions in the blade width of 10% and 3% for early and late WL, respectively, compared to WW, and reductions of 16% and 10% for blade length under the early and late WL treatments, respectively. Whereas all of these traits showed similar levels under both WL treatments, GW and senescence showed significant differences between the early and late drought: GW increased with drought level by 17% and 9% for the early and late WL, respectively, and canopy senescence increased by 62% and 38% for the early and late, respectively (Fig. 5). Heading was recorded about 4 d earlier (smaller HEA, 6%) under early WL relative to WW, whereas late WL did not differ from WW, as was expected since late WL was only initiated at 52 DAP. The number of spikes per plant was also reduced by early but not by late WL (14%). The drought treatment showed no significant effect on grain number (Fig. 5).

#### Productivity-related traits

**Fig. 6.**
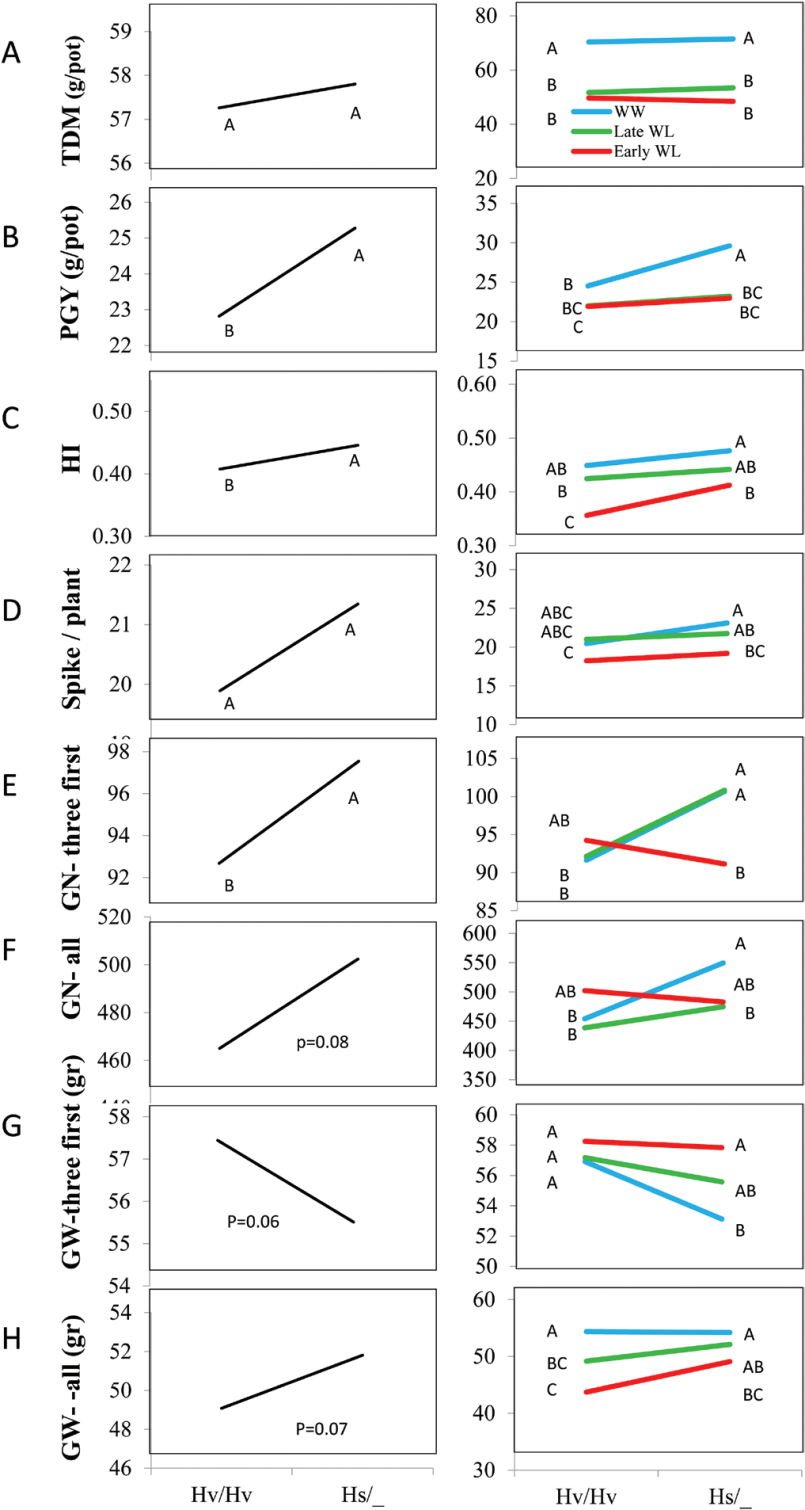
Genotype (left) and genotype × environment (right) interaction plots of HsDry2.2 for productivity-related traits. (A) TDM, total dry matter per plant (g), (B) PGY, plant grain yield (g), (C) HI, harvest index, (D) spikes per plant, (E) GN-three first, grain number of first three spikes (), (F) GN-all, grain number of all spikes, (G) GW-three first, grain weight of first three spikes (g), and (H) GW-all, grain weight of all spikes (g). Hv/Hv, homozygous for the Barke cultivated allele; Hs/_, homozygous or heterozygous for the wild HID062 allele. Different letters indicate significant differences at P<0.05 (Student’s t-test).

The genomic interval that defines HsDry2.2 as a Q×E QTL (see above, delimited by SCRI_RS_144592 and SCRI_RS_165574 at 54.2–59.9 cM) is a region of suppressed recombination, so markers are linked. It includes large number of markers in full linkage, e.g. SCRI_RS_208320, BOPA2_12_30265, and SCRI_RS_1249,2 and the *HvCEN* gene that resides between them (Maurer *et al.*, 2015). Therefore, genotyping the segregating BC_2_S_1_ with BOPA2_12_30265 is expected to give identical genotype-to-phenotype analysis to each of these loci. After genotyping the BC_2_S_1_ plants for the three genotypic groups (Supplementary Fig. S1), they were compared for their mean traits values. A dominant mode of inheritance was observed for most traits with no significant differences between the Hs/Hs and Hs/Hv genotypes (Tukey–Kramer test, *P*=0.05), for example mean spike dry weights were 30.8, 32.8, and 35.6 g for Hv/Hv, Hs/Hs, and Hv/Hs, respectively. We therefore decided to group the carriers of one or two wild alleles as single groups (hereafter Hs/_) to increase the number of replicates. TDM did not differ between Hs/_ and the cultivated (Hv/Hv) groups averaged across treatments and under each treatment separately (Fig. 6A). However, the wild allele conferred an advantage in PGY averaged across treatments (Fig. 6B), and this advantage was most pronounced under the WW treatment. The harvest index of the wild-allele carriers was significantly higher than in the cultivated allele group, especially under early WL (Fig. 6C). Spike number per plant did not differ significantly between genotypes under the different treatments, or averaged across treatments (Fig. 6D). The first three spikes to emerge were weighed and threshed separately, and their grain number (GN) was significantly higher in Hs/_ than in the Hv/Hv group (Fig. 6E). The genotype × treatment interaction (reaction norm) showed that this effect was obtained under both late WL and WW conditions, but not under early WL. The GN of all spikes was on average higher (+8%) for Hs/_ across treatments, and under the WW treatment (Fig. 6F). However, probably owing to high environmental variation the *P*-value was only of marginal significance (*P*=0.08), although we still consider this to be relevant as it is typical for such agricultural reproductive traits. In addition, while the wild allele was associated with lower GW of the first three spikes as compared to the cultivated one (mainly affected by the WW treatment, –3%, *P*=0.06; Fig. 6G), an opposite trend was observed for GW of all spikes (mainly having an increasing effect in the early WL treatment, +5.5%, *P*=0.07; Fig. 6H).

#### Phenology

**Fig. 7.**
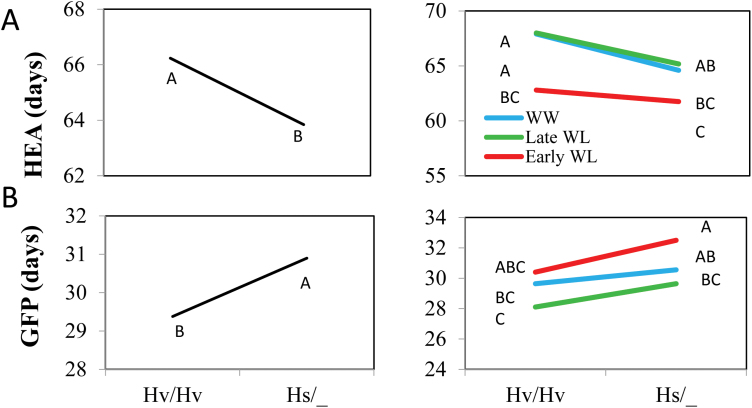
Genotype (left) and genotype × environment (right) interaction plots for phenological traits. (A) HEA, days from planting to heading; (B) GFP, grain filling period from HEA to maturity (d). Hv/Hv, homozygous for the Barke cultivated allele; Hs/_, homozygous or heterozygous for the wild HID062 allele. Different letters indicate significant differences at P<0.05 (Student’s t-test).

The first three spikes to emerge were used to determine plant phenology. The Hs/_ group emerged significantly earlier than the Hv/Hv plants (by 2.5 d; Fig. 7A), and this was more pronounced under WW and late WL than under early WL. The wild-allele carriers presented an elongated grain filling period across all treatments as compared to the cultivated allele (by 2 d; Fig. 7B).

#### Canopy structure

**Fig. 8.**
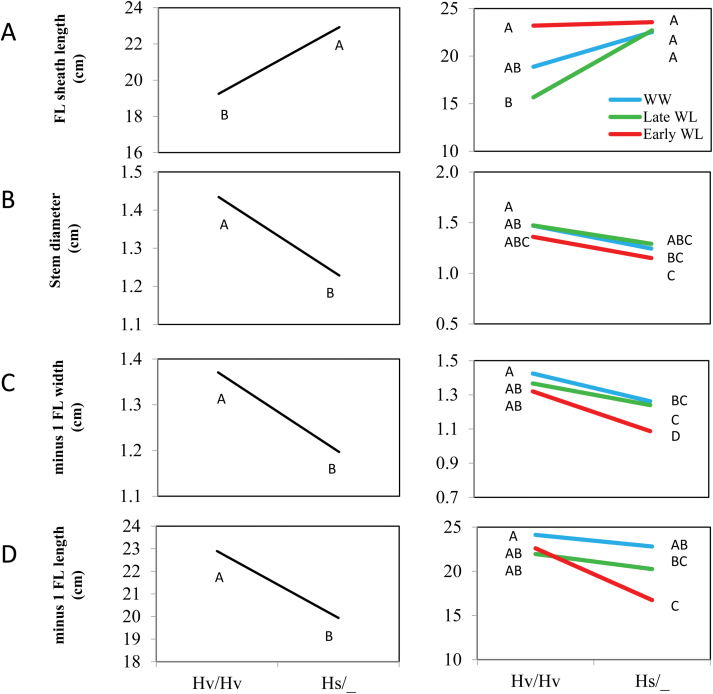
Genotype (left) and genotype ×environment (right) interaction plots for canopy structure traits. (A) FL (flag leaf) sheath length (cm), (B) stem diameter (cm), (C) minus 1 FL width, leaf blade width of the leaf preceding the flag leaf (cm), (D) minus 1 FL length, leaf blade length of the leaf preceding the flag leaf (cm). Hv/Hv, homozygous for the Barke cultivated allele; Hs/_, homozygous or heterozygous for the wild HID062 allele. Different letters indicate significant differences at P<0.05 (Student’s t-test).

Flag-leaf sheath length was higher in the Hs/_ group (Fig. 8A and Supplementary Fig. S5A). The wild-allele effect was very stable for this parameter across treatments, and the strongest differences between genotypes were observed under late WL. The Hs/_ plants exhibited constitutively reduced stem diameter (Fig. 8B and Supplementary Fig. S5B). The wild allele was generally associated with longer and narrower leaves; both the leaf blade width and length of the leaf preceding the flag leaf (–1FL) were found to be lower for wild-allele carriers, with the most pronounced difference being observed under early WL (Fig. 8C, D and Supplementary Fig. S5A).

#### Leaf senescence

**Fig. 9.**
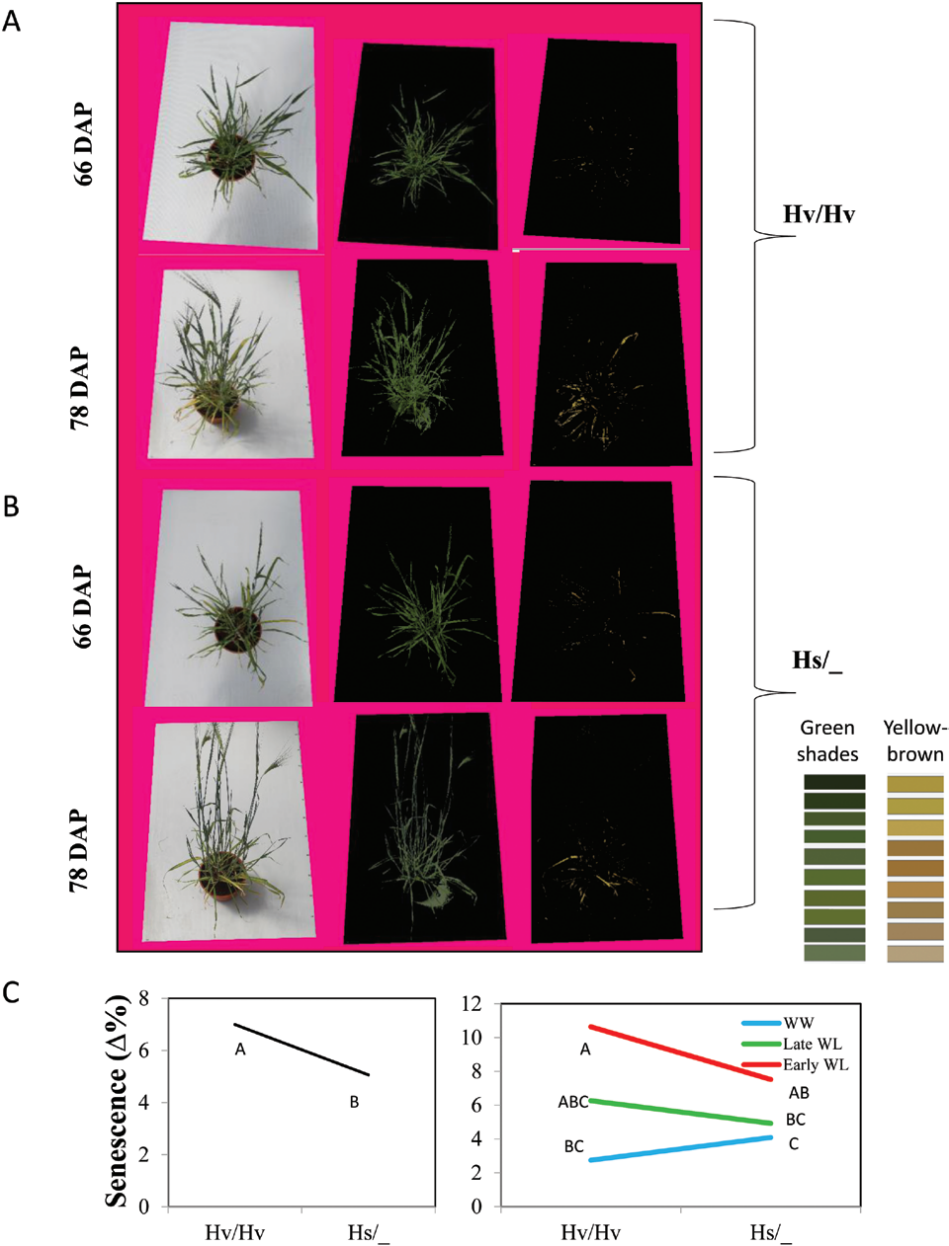
The HsDry2.2 locus is associated with reduced leaf senescence under drought. (A) Representative images used for analysis of senescence (early water-limited, WL), which was measured as the difference in the proportion of yellow-brown areas out of the total canopy area between two sets of images taken at 66 and 78 d after planting (DAP). The analysis was conducted using a custom-made software. (B) Genotype (left) and genotype × environment (right) interaction plots for leaf senescence. Hv/Hv, homozygous for the Barke cultivated allele; Hs/_, homozygous or heterozygous for the wild HID062 allele. Different letters indicate significant differences at P<0.05 (Student’s t-test).

Analysis of senescence was carried out by determining the difference in the area of yellow-brown coloration out of the total canopy area over a period of 12 d (66–78 DAP, Fig. 9A, B). No significant differences were found in total canopy area between the genotypic groups at both the dates, nor in the ratio of yellow-brown/total at 66 DAP (data not shown). However, at 78 DAP the carriers of the cultivated allele exhibited on average a significantly higher yellow-brown area out of total leaf area as compared to the Hs/_ group. Greater senescence, evaluated by calculating the differences between dates, was observed for Hv/Hv (Fig. 9C). These differences in senescence between the two genotypic groups were notably more pronounced under both drought-stress treatments (Fig. 9C).

## Discussion

### The HEB-25 genetic architecture for flowering time

The genetic architecture for flowering time identified in our experiments was similar to that reported previously by Maurer *et al.* (2015) and Saade *et al.* (2016), with the exception of *Vrn-H2* that was not identified in our study. The main loci for HEA, including *HvELF3*, *Ppd-H1*, *HvCEN*, *denso*, *Vrn-H1*, and *Vrn-H3*, were identified (Fig. 2 and Supplementary Table S4). Nevertheless, an interesting difference between the experiment we conducted in Israel (E34°47′, N31°54′) and previous experiments conducted in Germany (E11°58′, N51°29′) was the effect of *Ppd-H1* on HEA. Unlike the earliness effect of the wild allele seen in Germany, namely a mean reduction of 9.5 d in heading time (Maurer *et al.*, 2015), under our conditions an opposite effect was observed, with an increase of 6.7 d in mean heading time under both the growth conditions that we used. This effect is similar to that reported in another study using the same HEB-25 population by Saade *et al.* (2016), and to that found in other studies (Pan *et al.*, 1994; Borras-Gelonch *et al.*, 2012; Ponce-Molina *et al.*, 2012). In all these populations, a QTL for heading date was detected in the region of *PpdH1*, but the phase and the magnitude of the effects varied depending on the environment, which highlights the role of this gene in photoperiod sensitivity (Turner *et al.*, 2005). These types of opposite effects should be taken into account when '‘designing’ an ideotype or by accumulating several independent traits in the same genotype through introgression breeding of wild QTLs (pyramiding ; Zamir, 2001): these types of QTLs originating from wild alleles could be beneficial in one environment and detrimental in another.

### Genome scan for Q×E interactions

In this study, a genome scan was performed to search for loci with significant Q×E interactions, as manifested by environmentally conditioned differences in mean trait measures between the carriers of the wild allele (Hs/Hs) as compared to plants homozygous for the cultivated allele (Hv/Hv). This approach was different from recent Q×E studies in Arabidopsis, in which analyses were performed in two stages: initially performing a genome scan for loci that are associated with the trait variation *per se* regardless of the environment, mainly in order to maximize QTL identification, and only then testing for the Q×E interactions for these loci (El-Soda *et al.*, 2014). In addition, it was different from the way genotype × environment interaction are often treated as a variation component that would improve the percentage variation explained by the genetic model (Elias *et al.*, 2016). HsDry2.2 is an excellent example of a major Q×E locus (Fig. 3) for reproductive output traits (PGY and GN) that was not identified by GWAS for the trait *per se* in either WW or WL conditions (Fig. 2, Supplementary Tables S4, S5). However, when the genome scan was screened for Q×E, it was highlighted as the most reproducible Q×E locus with similar effects observed over two years of field trials.

### Prevalence of Q×E QTL for reproductive rather than vegetative or phenological traits

In this study we were able to identify 69 loci for traits *per se* and 22 for Q×E (Fig. 2, Supplementary Tables S4, S5). In general, comparison of the Q×E genomic architecture to that of the trait variations *per se* showed that there were very few Q×E loci for some of the traits. Moreover, some bias for reproductive trait-associated Q×E loci was apparent (Supplementary Tables S4, S5). This did not seem to be the result of the limited variation of vegetative or phenological traits in the HEB-25 lines under our experimental set-up (Fig. 1A).

Interestingly, the prevalence of conditional QTLs was far higher for PGY and GN as compared to other traits, in which the large number of QTLs for trait *per se* was dramatically reduced when considering interactions. Looking more carefully at the reaction norms of such QTLs (Fig. 4 and Supplementary Fig. S3) showed that the carrier of the wild allele experienced less reduction under WL, rather than simply increasing the PGY or GN (i.e. the wild allele is conferring phenotypic stability under changes in the watering regime). Although this was only being observed for a semi-controlled environment and it requires validation under a more realistic agricultural set-up, such a locus has a promising potential to provide grain yield stability against drought. Future experiments therefore need to test isogenic lines for this wild allele in several genetic backgrounds, and with larger plots.

It has been argued that since drought survival is a trait under strong evolutionary pressure, many drought survival loci would be expected to impart tolerance in crop plants by accumulation of small-effect QTLs (Mickelbart *et al.*, 2015). However, when considering grain yield and the exploitation of interspecific crosses, such as the HEB-25 population, one caveat should be considered. Wild relatives of modern crops adapt to drought not necessarily by producing more grains under stress, a strategy that might be detrimental for maintenance of wild populations under limited and fluctuating resources. Instead, upon dissection of the wild genome and examination of its parts in a cultivated genetic background, as in our study, we would not necessarily expect to find alleles from the wild that would increase grain number and yield under stress. Rather, the alleles that we would expect to find are ones that stabilize or buffer the effects of the stressful environment on the plant. For example, owing to the wide allelic variation existing in the HEB-25 population, we were able to identify several significant Q×E loci, including a major locus that implicates a hitherto unknown role for a ‘flowering time gene’ in regulating drought resilience in what seems as a non-escape mechanism. The pot experiment showed a similar trend of shortening time to flowering (only by ~2.5 d) of the wild allele as compared to the cultivated one. The much earlier general heading date in the pot experiment (~65 d), as compared to the field trials (~100 d), was probably related to differences in thermal day-degrees between the experiments (Supplementary Fig. S6). Hence, in the relatively hot climate of the pot experiment the plants flowered earlier, probably as an effect of the different environmental conditions, and thus the differences between them were seen to a lesser extent than in the field. Furthermore, under these hot conditions both genotypes flowered early, ruling out a drought-escape mechanism of one allele compared to the other. Alternatively, the earlier flowering and elongated grain filling of the wild allele may in part underlie its improved productivity.

### Flowering-independent Q×E effects of HsDry2.2 on reproductive traits

The most reproducible and significant Q×E locus was identified on the long arm of chromosome 2H (Figs 2, 3). Interestingly, this position matches the location of *HvCEN*, the barley ortholog of the *CENTRORADIALIS* gene, with two main haplotypes differentially distributed across spring and winter barley varieties (Comadran *et al.*, 2012). Under WL conditions, HsDry2.2 showed conditional effects on the total plant grain number, but no such interaction with flowering time (Fig. 4). Cuesta-Marcos *et al.* (2009) found QTLs for HEA, as well as for Q×E for grain yield, in the region of *Eam6/HvCEN* in a barley Beka × Mogador population. In a Nure × Tremois population, Francia *et al.* (2011) found *Eam6* to explain only ~6% of the genotype × environment (the latter representing different water availabilities) variance, as compared to 35% of the genotype component.

Both field trials and pot experiment showed that the wild allele had no advantage over the cultivated one in vegetative or TDM production; however, it showed superiority for reproductive traits (PGY and HI). Recently, loci regulating developmental characteristics were found to be co-located with flowering-time genes, including *HvCEN* (Maurer *et al.*, 2016; Nice *et al.*, 2017); however, this locus was not associated with height differences (Nice *et al.*, 2017). Maurer *et al.* (2016) found significant effects of ‘QTL-2H-7’ (with *HvCEN* as the candidate gene) on all measured traits; however, they did not measure yield. It would therefore be interesting to further characterize these differences at the metabolic and developmental levels, both in the sink and source tissues, under gradients of water availability for isogenic lines for this Q×E locus. This may lead to the identification of hitherto unknown pathways related to drought tolerance.

### HsDry2.2 effects under early versus late drought

Despite the similarity in reduced PGY for the early and late WL treatments, it was quite clear than the reduction was obtained via different pathways. As expected, while spikes per plant was significantly reduced by early WL, in the late WL treatment it was not. Total GW (i.e. all spikes) under late WL was reduced by only half of that of early WL, and GN appeared to be reduced only in the early WL treatment, although not significantly (Supplementary Table S6).

As temperatures in the greenhouse were high throughout the entire season (Supplementary Fig S6), it is reasonable to assume that all plants were under mild heat stress. In addition, as plant water demand grew towards the middle of the season, the field water capacity dropped below 60% in the WW treatment when irrigation was once every two days (and not daily). Therefore, the advantage in PGY for the wild allele that was obtained mainly under WW might also reflect heat resistance.

Whereas under the WW treatment the benefit of the wild allele on PGY may be attributed to higher GN, under both the early and late WL treatments the wild allele presented advantages in GW, ripening period, and reduced senescence rate. In that respect, the condensed canopy structure of the drought-adapted wild allele under early WL may also have had an effect on the reduced senescence rate as a ‘by-product’ of this phenotype.

Overall, the Hs/_ plants presented a phenotype that matched the desired ideotype of future climate-resilient barley for Mediterranean climatic zones based on crop models (Tao *et al.*, 2017), i.e. a longer reproductive growing period (similar to longer GFP), lower leaf senescence rate, and higher drought tolerance. This indicates the importance of this QTL for future breeding of barley.

Comadran *et al.* (2012) reported three major haplotypes of HvCEN shared between wild and cultivated barleys. Haplotypes I (Morex) and III (Tremois) both carry Ala135 (G in the conserved position), but differ in two SNPs in intron 2. Haplotype II (Nure) is the one that has a C giving rise to Pro135. Notably, resequencing this SNP in the HEB-04 and HEB-05 families, including HEB-04-096 that served as the source for the segregating BC_2_S_1_ plants in our pot experiment, showed that the Hs and Hv alleles of the HEB-04 family differ at the position that Comadran *et al.* (2012) termed Pro135A, and at an additional SNP at the 3′-untranslated region (Supplementary Fig. S7, Table S1). However, these non-synonymous and synonymous SNP variants, respectively, were not shared with plants of the HEB-05 family carrying introgression from the wild at this position (Supplementary Fig. S7, Table S1). The lack of segregating SNPs at *HvCEN* in the HEB-05 family may indicate that the casual variation underling the improved drought resistance of the Hs allele over the Hv one in this study may be attributed to variations other than the C-to-G SNP substitution underlying P135A (Comadran *et al.*, 2012), i.e. that there is another variation shared by HEB-04 and HEB-05 that cause these effects. Moreover, it may imply that there could be causal genes other than *HvCEN* itself, which is locked within this chromosomal region, as was shown recently (Mascher *et al.*, 2017), and therefore dissecting the true causal variation in this region remains a challenge

Overall, the current study provides data from large-scale genome-wide association scanning to a finer-resolution scale, and sheds some more light on the promising HsDry2.2 locus and its mode of action. This appears to be a rare case of a wild allele with direct beneficial effects on grain yield. Finer mapping of this locus should determine whether the causal gene for these multiple differences between the wild and cultivated alleles does indeed correspond to *HvCEN*, or whether a nearby previously overlooked gene (or genes) is responsible.

## Supplementary data

Supplementary data are available at *JXB* online.

Table S1. Primers used for high-resolution melt (HRM) and HvCEN sequencing.

Table S2. Trait distributions and heritability values under WW and WL treatments.

Table S3. Pairwise correlations between traits under WW and WL treatments in the field.

Table S4. GWAS results for traits *per se*.

Table S5. Q×E loci for the different traits.

Table S6. Pot experiment ANOVA for measured traits.

Fig. S1. Genotyping for the BC_2_F_1_ pot experiment.

Fig. S2. Details of the irrigation management in the pot experiment.

Fig. S3. Reaction norms for vegetative dry matter in the field experiment.

Fig. S4. Reaction norms for grain number for HEB-05 and HEB-16 families in the field experiment.

Fig. S5. Shoot morphology in the pot experiment for Hv/Hv and Hs/_.

Fig. S6. Temperature profile for the pot experiment.

Fig. S7. Sequencing results for *HvCEN*.

File S1. The in-house R script utilizing the GWAF and kinship2 R-packages.

Supplementary Fig S1-S4

Supplementary Fig S5-S7

supplementary Tables

Supplementary figure legends

## Author contributions

RS, LOM, EL, and EF designed the experiments, collected the data, conducted the data analysis and interpretations, and wrote the manuscript. RS, AF, and LOM were involved in the data analysis and its interpretation, and assisted in writing the manuscript. KP and AM supplied the plant material and genotype data.
