# Supplementary material for: Genome scan identifies flowering-independent effects of barley HsDry2.2 locus on yield traits under water deficit
Source: J Exp Bot. 2018 Jan 8;69(7):1765–79. doi: 10.1093/jxb/ery016 (PMC5888960; doi:10.1093/jxb/ery016)
Supplement: Supplementary Fig S1-S4 [file ery016_suppl_supplementary_fig_s1-s4.pptx]

## Slide 1
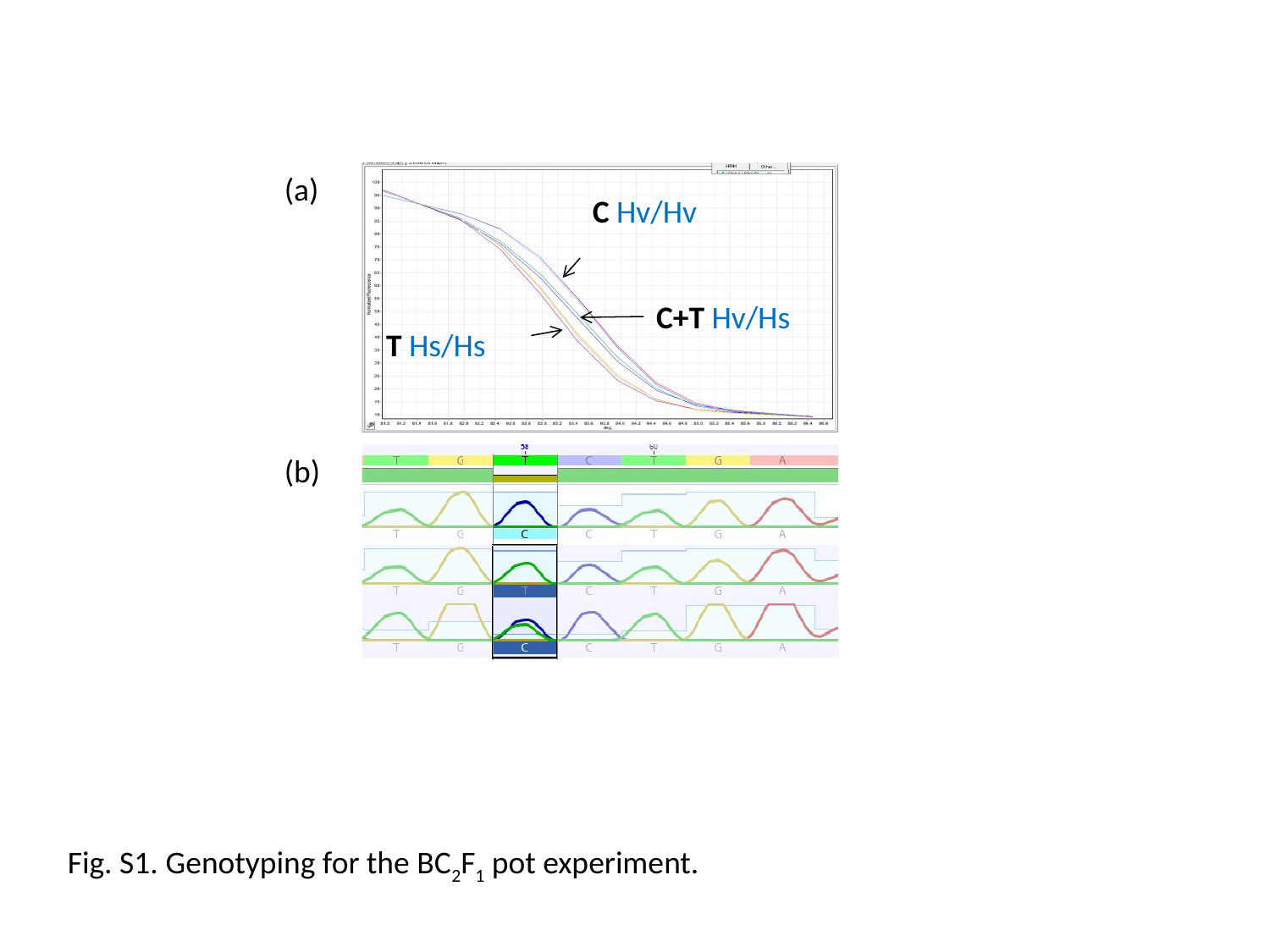

(a)
C Hv/Hv
C+T Hv/Hs
T Hs/Hs
(b)
Fig. S1. Genotyping for the BC2F1 pot experiment.

## Slide 2
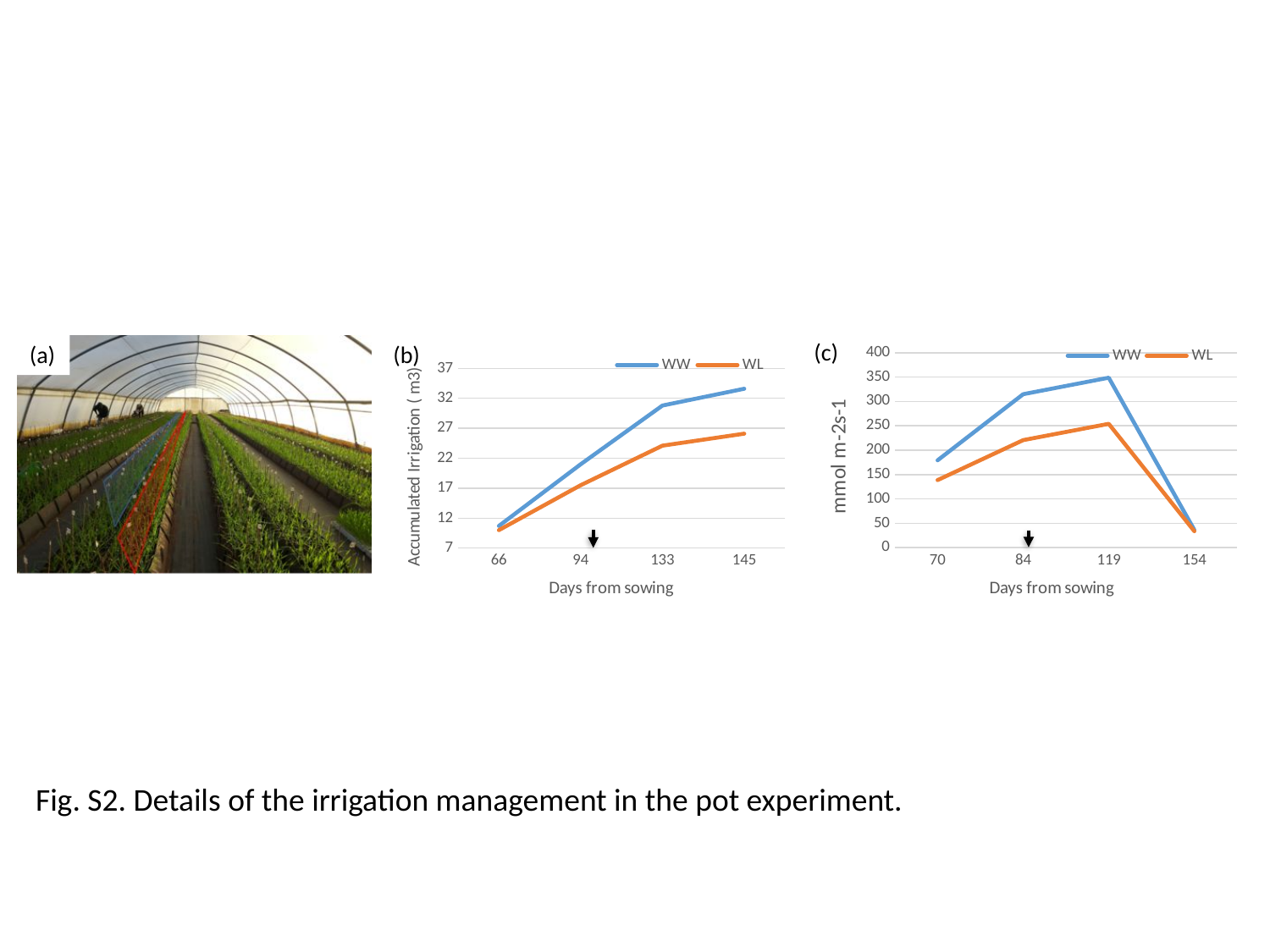

### Chart
| Category | WW | WL |
|---|---|---|
| 70.0 | 179.25 | 138.5375 |
| 84.0 | 314.8374999999999 | 220.675 |
| 119.0 | 348.625 | 254.0375 |
| 154.0 | 36.4 | 33.8 |
### Chart
| Category | WW | WL |
|---|---|---|
| 66.0 | 10.7 | 10.0 |
| 94.0 | 21.0 | 17.5 |
| 133.0 | 30.8 | 24.1 |
| 145.0 | 33.6 | 26.1 |(c)
(a)
(b)
Fig. S2. Details of the irrigation management in the pot experiment.

## Slide 3
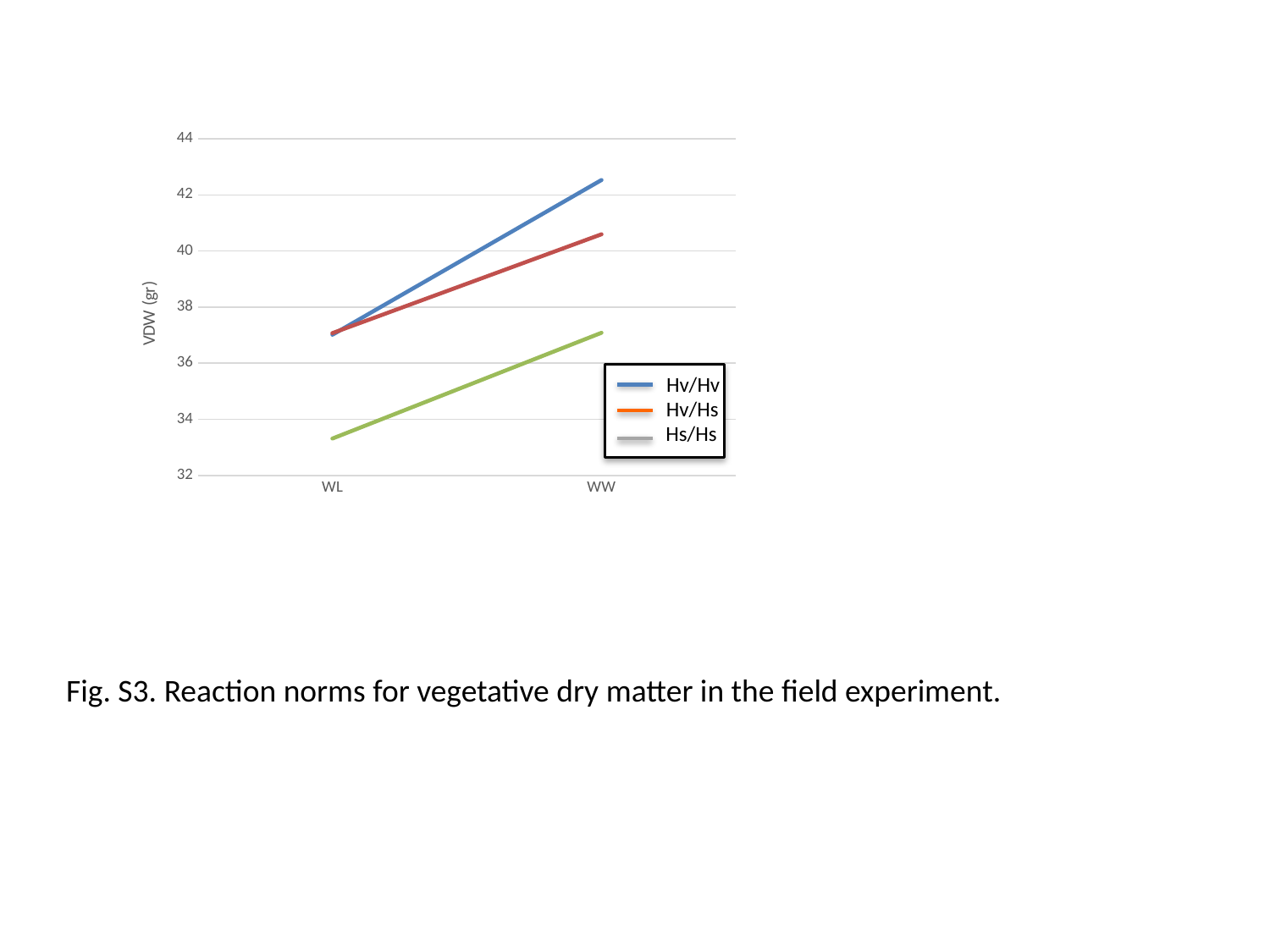

### Chart
| Category | Hv/Hv | Hv/Hs | Hs/Hs |
|---|---|---|---|
| WL | 37.01270400000001 | 37.07137100000001 | 33.313633 |
| WW | 42.52895900000001 | 40.59590700000001 | 37.083421 |
Hv/Hv
Hv/Hs
Hs/Hs
Fig. S3. Reaction norms for vegetative dry matter in the field experiment.

## Slide 4
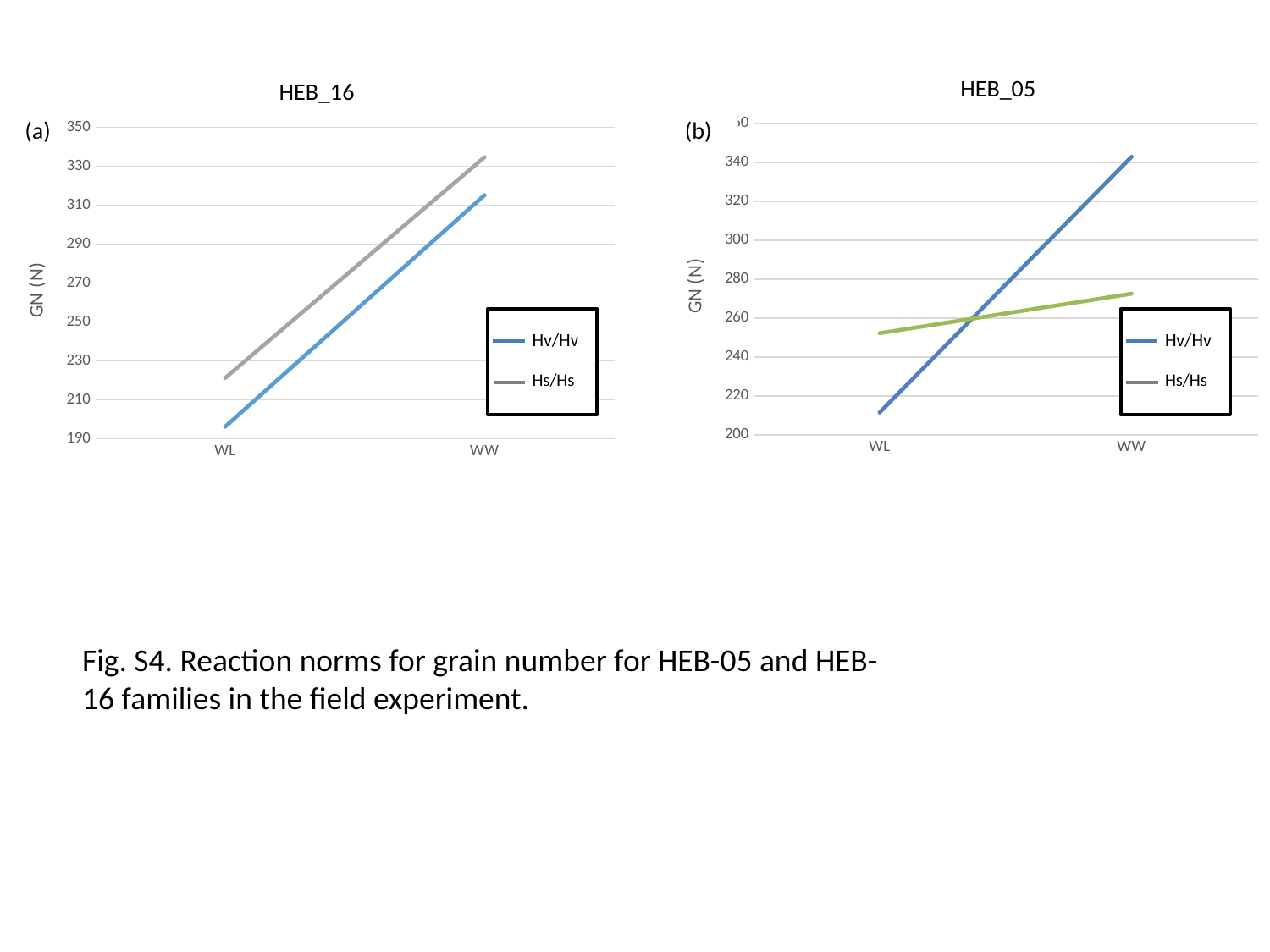

HEB_05
HEB_16
[unsupported chart]
(a)
(b)
[unsupported chart]
Hv/Hv
Hs/Hs
Hv/Hv
Hs/Hs
Fig. S4. Reaction norms for grain number for HEB-05 and HEB-16 families in the field experiment.
