# Supplementary material for: Genome scan identifies flowering-independent effects of barley HsDry2.2 locus on yield traits under water deficit
Source: J Exp Bot. 2018 Jan 8;69(7):1765–79. doi: 10.1093/jxb/ery016 (PMC5888960; doi:10.1093/jxb/ery016)
Supplement: Supplementary Fig S5-S7 [file ery016_suppl_supplementary_fig_s5-s7.pptx]

## Slide 1
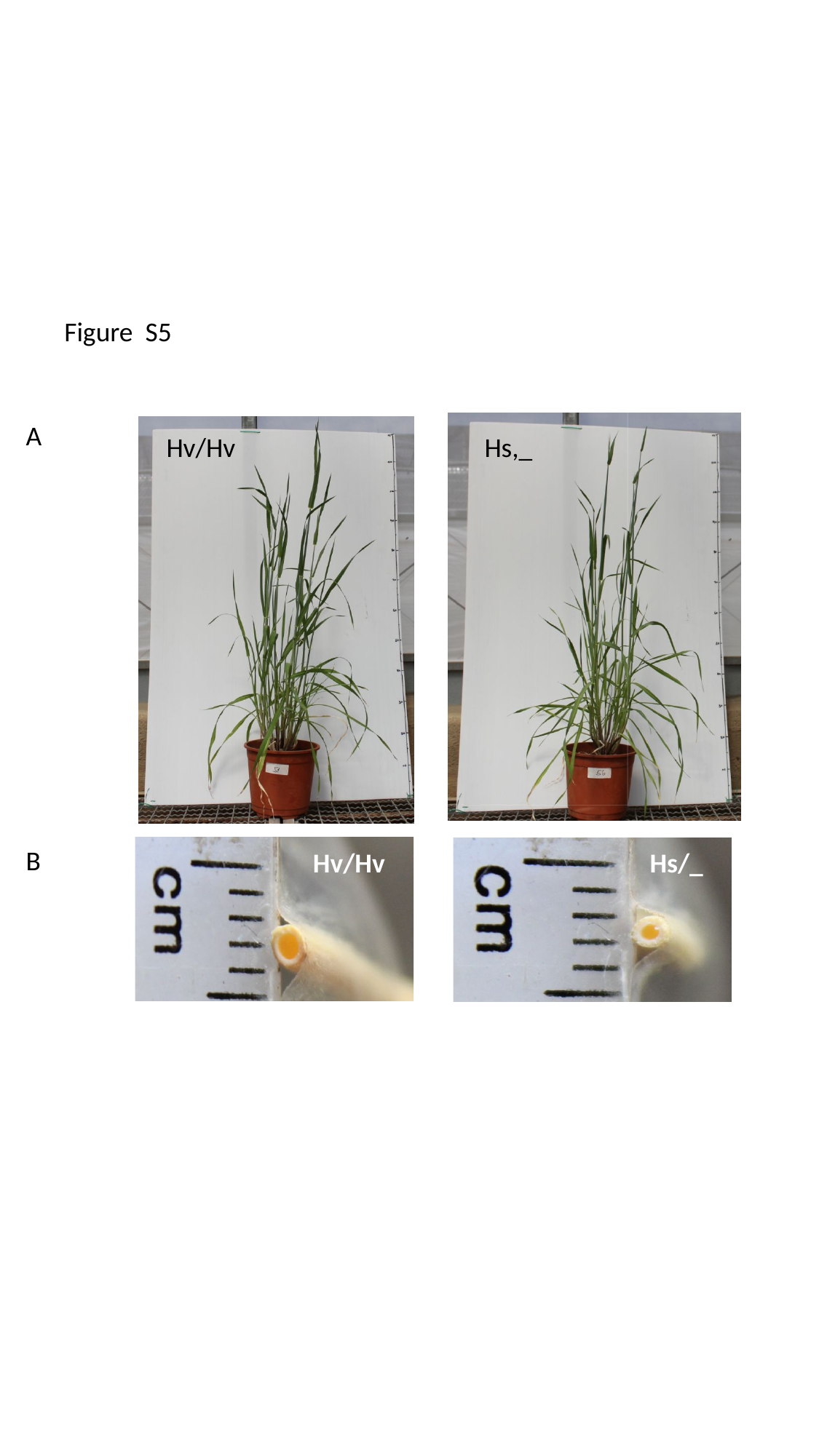

Figure S5
A
Hv/Hv
Hs,_
B
Hv/Hv
Hs/_

## Slide 2
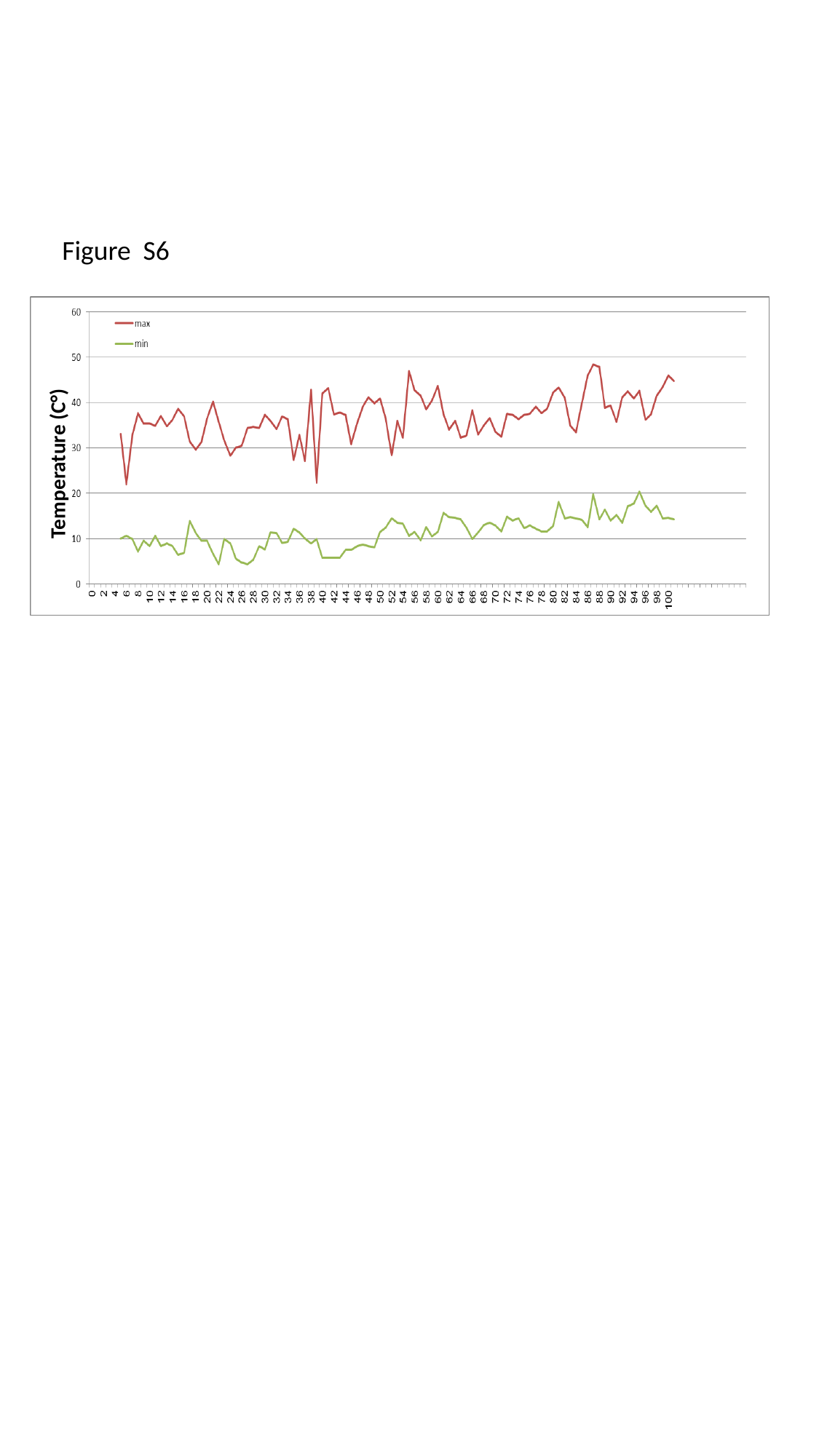

Figure S6
Temperature (C°)

## Slide 3
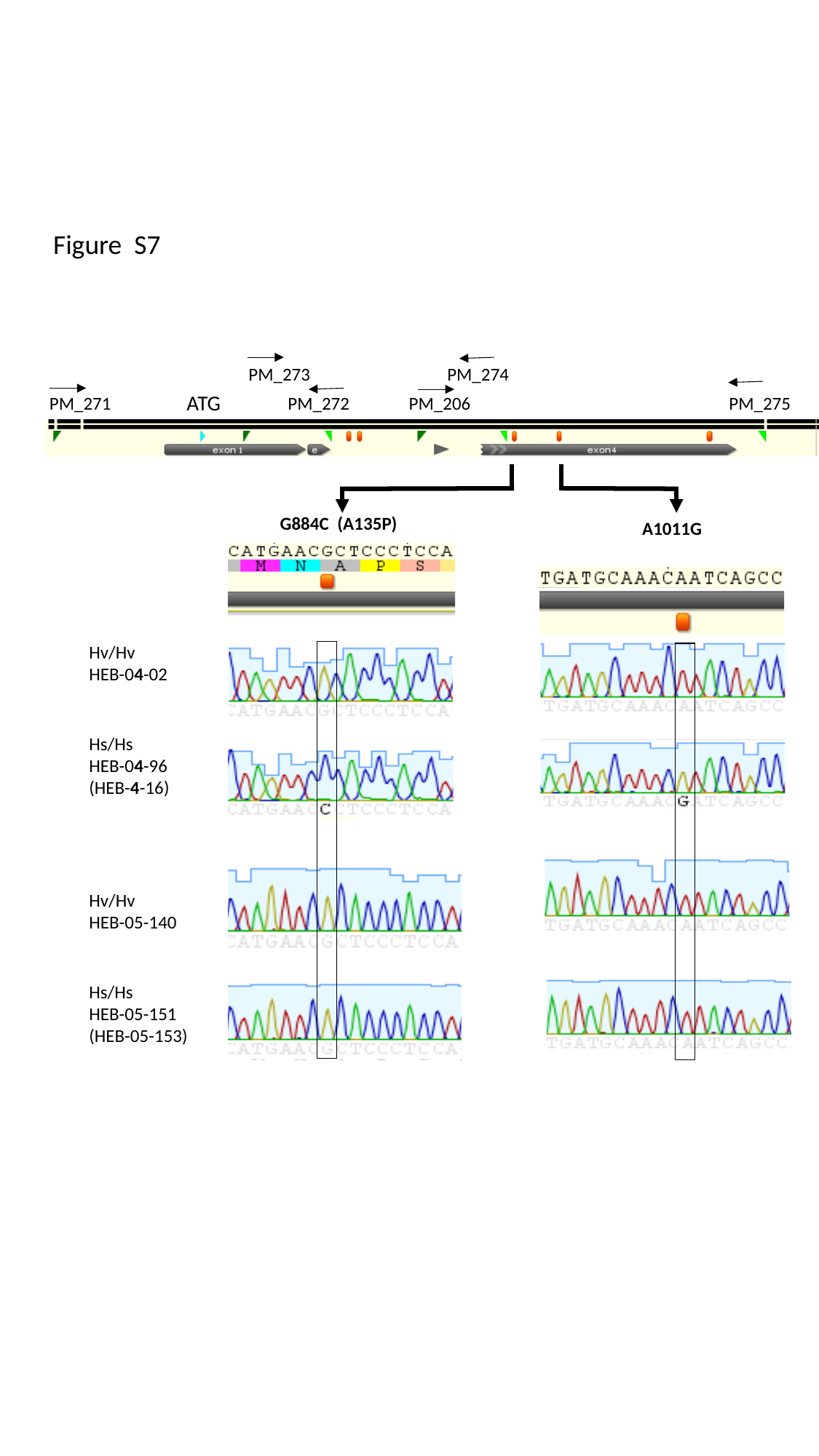

Figure S7
PM_273
PM_274
ATG
PM_271
PM_272
PM_206
PM_275
G884C (A135P)
A1011G
Hv/Hv
HEB-04-02
Hs/Hs
HEB-04-96
(HEB-4-16)
Hv/Hv
HEB-05-140
Hs/Hs
HEB-05-151
(HEB-05-153)
