# Supplementary material for: Genome scan identifies flowering-independent effects of barley HsDry2.2 locus on yield traits under water deficit
Source: J Exp Bot. 2018 Jan 8;69(7):1765–79. doi: 10.1093/jxb/ery016 (PMC5888960; doi:10.1093/jxb/ery016)
Supplement: supplementary Tables [file ery016_suppl_supplementary_tables.pdf]

Supplementary data

Manuscript Title: Genome scan identifies flowering-independent effects of barley HsDry2.2 locus on yield traits under water deficit **Authors:** Lianne Merchuk-Ovnat*, Roi Silberman*, Efrat Laiba, Andreas Maurer, Klaus Pillen, Adi Faigenboim, Eyal Fridman

Table S1 Primers used for HRM and HvCEN sequencing

Primer name		Use	sequence
PM58	F	HRM for BOPA2_12_30265	AGGTTTCCCTCGGGTTCAG
PM59	R	HRM for BOPA2_12_30265	GGCAGGGTACGTCTGTCTCT
PM206	F	sequencing *HvCEN* (HORVU2Hr1G072750)	AGCTATGCGTAAAAACATGCTTGA
PM271	F	sequencing *HvCEN* (HORVU2Hr1G072750)	TGTAGGCGCATACATGGCAA
PM272	R	sequencing *HvCEN* (HORVU2Hr1G072750)	GGTTACCAGTGGAGGTGCTC
PM273	F	sequencing *HvCEN* (HORVU2Hr1G072750)	AATGGCCACGAGTTCTTCCC
PM274	R	sequencing *HvCEN* (HORVU2Hr1G072750)	TGTTGGAACAGCACGAAGGT
PM275	R	sequencing *HvCEN* (HORVU2Hr1G072750)	ACATGGAGGGAGGGAGAACA

Supplementary data

Manuscript Title: Genome scan identifies flowering-independent effects of barley HsDry2.2 locus on yield traits under water deficit

Authors: Lianne Merchuk-Ovnat*, Roi Silberman*, Efrat Laiba, Andreas Maurer, Klaus Pillen, Adi Faigenboim, Eyal Fridman

Table S2 Traits distribution and heritability values under WW and WL environments

h2, Broad-sense heritability calculated as proportion of variance explained by genotype across two years

Year	Trait	Units	WW						WL						
			N	Mean	Min	Max	STD	cv	N	Mean	Min	Max	STD	cv	WL/WW
2015	GFP	Days	1399	36	15	58	6.4	17.9	1359	29.7	10	60	7	23.6	0.8
2015	GN	#	1294	297	34	681	104	34.9	1000	204.7	3	710	135	66.1	0.7
2015	GW	Gram	1292	66.5	23	227	16.1	24.2	995	37.5	0.2	125	18.2	48.6	0.6
2015	PGY	Gram	1413	17.8	0.1	41	6.3	35.1	1029	7.5	0.1	33.4	6.7	89.1	0.4
2015	HEA	Days	1429	97.1	80	120	7.6	7.8	1407	96.4	81	118	7.6	7.9	1
2015	HEI	cm	1428	72.8	45	126	12.9	17.7	1424	63.2	31	120	12.8	20.3	0.9
2015	HI	%	1385	31	0.1	69	9.9	32.1	1381	13.5	0.1	56.6	15	111	0.4
2015	MAT	Days	1403	133	101	152	6.9	5.2	1383	125.5	108	157	7.4	5.9	0.9
2015	TDM	Gram	1387	59.6	15	118	15	25.2	1382	42.1	10	112	13.3	31.5	0.7
2015	VDW	Gram	1371	41.2	5.2	80	13.2	32.1	1372	36.2	7	79.9	12.8	35.4	0.9
2016	GFP	Days	1280	33.3	10	64	5.4	16.2	1273	30.1	15	64	6.7	22.4	0.9
2016	GN	#	1156	300	26	730	93.4	31.1	1136	180	4	562	89.4	49.7	0.6
2016	GW	Gram	1147	46.2	14	72	6.0	13.0	1111	41.9	3	92	7.5	17.8	0.9
2016	PGY	Gram	1277	13.9	0.9	30	4.3	31.3	1250	7.6	0.1	20.9	3.9	50.9	0.5
2016	HEA	Days	1284	104	74	124	8.3	8	1294	102.2	74	125	8.7	8.5	1
2016	HEI	cm	1295	70.2	40	115	11.5	16.4	1291	59.1	30	98	10.9	18.4	0.8
2016	HI	%	1270	30.8	2.7	65	8.1	26.3	1245	22	0.3	73.6	10.3	46.7	0.7
2016	MAT	Days	1281	137	120	149	6.5	4.8	1274	132.2	100	149	5.9	4.5	1
2016	TDM	Gram	1289	45.2	11	83	10	22	1287	34.8	13.6	71.4	9.3	26.7	0.8
2016	VDW	Gram	1270	31.5	11	66	8.2	26	1243	27.3	5.7	58.7	8.1	29.6	0.9

Year	Trait	Units	WW							WL							WL/ WW
			N	Mean	Min	Max	STD	cv	h2	N	Mean	Min	Max	STD	cv	h2	
15&16	GFP	Days	1296	36	19	61	4.9	13.6	0.62	1292	30.1	11.1	52.3	5.6	18.6	0.68	0.8
15&16	GN	#	1295	301	48	686	79.2	26.4	0.69	1244	207	5.7	583	96.4	46.5	0.55	0.7
15&16	GW	Gram	1453	66.4	29	175	11.2	16.9	0.71	1453	41.0	0	92	11.2	27.3	0.52	0.6
15&16	GY	Gram	1296	18.3	7	32	4.1	22.1	0.72	1283	7.6	0.1	24.9	4.1	53.2	0.55	0.4
15&16	HEA	Days	1296	103	82	125	7.5	7.3	0.6	1294	102	80.9	122	7.7	7.5	0.65	1
15&16	HEI	cm	1296	72.6	47	110	10.1	14	0.63	1295	63.2	37.6	101	9.9	15.7	0.62	0.9
15&16	HI	%	1254	31.6	7.3	57	6.8	21.5	0.75	1220	22.2	0.3	66	13.7	61.7	0.55	0.7
15&16	MAT	Days	1296	137	119	157	5.7	4.2	0.59	1292	132	121	153	5.7	4.3	0.66	1
15&16	TDM	Gram	1274	59.9	28	106	11.1	18.5	0.67	1265	42.2	16.7	79.1	9.4	22.3	0.63	0.7
15&16	VDW	Gram	1242	41.1	14	79	9.7	23.6	0.62	1212	36.2	14.8	66.1	9.1	25.1	0.59	0.9

Supplementary data
Manuscript Title: Genome scan identifies flowering-independent effects of barley HsDry2.2 locus on yield traits under water deficit
Authors: Lianne Merchuk-Ovnat*, Roi Silberman*, Efrat Laiba, Andreas Maurer, Klaus Pillen, Adi Faigenboim, Eyal Fridman

Table S3 Pairwise correlations between traits under WL and WW

WW/WL	Variable	by Variable	Correlation	Count	Lower 95%	Upper 95%	Signif Prob
WL	GN	GFP	0.2435	1242	0.1905	0.2952	<.0001
WL	GW	GFP	0.0412	1241	-0.0145	0.0966	0.1469
WL	GW	GN	0.0461	1243	-0.0095	0.1015	0.104
WL	PGY	GFP	0.2548	1281	0.2029	0.3053	<.0001
WL	PGY	GN	0.814	1240	0.7943	0.8319	<.0001
WL	PGY	GW	0.4321	1239	0.3856	0.4763	<.0001
WL	HEA	GFP	-0.6681	1292	-0.6972	-0.6368	<.0001
WL	HEA	GN	-0.2287	1244	-0.2807	-0.1754	<.0001
WL	HEA	GW	-0.0694	1243	-0.1245	-0.0138	0.0144
WL	HEA	PGY	-0.2349	1283	-0.2859	-0.1825	<.0001
WL	HEI	GFP	0.1117	1232	0.0562	0.1665	<.0001
WL	HEI	GN	0.0613	1187	0.0044	0.1178	0.0347
WL	HEI	GW	0.0452	1186	-0.0118	0.1018	0.12
WL	HEI	PGY	0.0748	1223	0.0188	0.1303	0.0089
WL	HEI	HEA	-0.1346	1234	-0.1889	-0.0794	<.0001
WL	HI	GFP	0.1575	1220	0.1022	0.2117	<.0001
WL	HI	GN	0.5542	1181	0.5134	0.5925	<.0001
WL	HI	GW	0.3901	1181	0.3406	0.4374	<.0001
WL	HI	PGY	0.7315	1220	0.7043	0.7565	<.0001
WL	HI	HEA	-0.193	1220	-0.2465	-0.1384	<.0001
WL	HI	HEI	-0.0033	1160	-0.0608	0.0543	0.9107
WL	MAT	GFP	0.1178	1292	0.0636	0.1712	<.0001
WL	MAT	GN	-0.0346	1242	-0.09	0.0211	0.2235
WL	MAT	GW	-0.0728	1241	-0.1279	-0.0172	0.0103
WL	MAT	PGY	-0.0344	1281	-0.089	0.0204	0.2188
WL	MAT	HEA	0.6225	1292	0.5879	0.6548	<.0001
WL	MAT	HEI	-0.0639	1232	-0.1194	-0.0081	0.0248
WL	MAT	HI	-0.0778	1220	-0.1334	-0.0218	0.0065
WL	TDM	GFP	0.1029	1265	0.0481	0.1572	0.0002
WL	TDM	GN	0.3432	1218	0.2927	0.3919	<.0001
WL	TDM	GW	0.0833	1217	0.0272	0.1388	0.0036
WL	TDM	PGY	0.3352	1255	0.2852	0.3835	<.0001
WL	TDM	HEA	-0.0609	1265	-0.1157	-0.0058	0.0302
WL	TDM	HEI	0.1832	1205	0.1281	0.2372	<.0001
WL	TDM	HI	-0.0689	1220	-0.1245	-0.0128	0.0161
WL	TDM	MAT	0.0179	1265	-0.0372	0.073	0.5245
WL	VDW	GFP	0.0016	1212	-0.0548	0.0579	0.9569
WL	VDW	GN	0.0189	1173	-0.0384	0.0761	0.5179
WL	VDW	GW	-0.0814	1173	-0.138	-0.0242	0.0053
WL	VDW	PGY	-0.054	1212	-0.11	0.0023	0.0601
WL	VDW	HEA	0.0531	1212	-0.0032	0.1091	0.0647
WL	VDW	HEI	0.158	1152	0.1012	0.2138	<.0001
WL	VDW	HI	-0.4759	1212	-0.5183	-0.4311	<.0001
WL	VDW	MAT	0.0603	1212	0.004	0.1162	0.0357
WL	VDW	TDM	0.8927	1212	0.8807	0.9036	<.0001

WW/WL	Variable	by Variable	Correlation	Count	Lower 95%	Upper 95%	Signif Prob
WW	GN	GFP	-0.1048	1295	-0.1583	-0.0506	0.0002
WW	GW	GFP	0.1147	1295	0.0606	0.1682	<.0001
WW	GW	GN	-0.4408	1295	-0.4837	-0.3958	<.0001
WW	PGY	GFP	-0.0402	1296	-0.0944	0.0143	0.1483
WW	PGY	GN	0.8115	1295	0.792	0.8293	<.0001
WW	PGY	GW	-0.0132	1295	-0.0676	0.0413	0.635
WW	HEA	GFP	-0.6285	1296	-0.6604	-0.5944	<.0001
WW	HEA	GN	0.096	1295	0.0417	0.1497	0.0005
WW	HEA	GW	-0.0236	1295	-0.078	0.0309	0.3964
WW	HEA	PGY	0.0855	1296	0.0312	0.1393	0.0021
WW	HEI	GFP	0.1705	1296	0.1172	0.2229	<.0001
WW	HEI	GN	-0.0664	1295	-0.1204	-0.0119	0.0169
WW	HEI	GW	0.0929	1295	0.0386	0.1466	0.0008
WW	HEI	PGY	-0.0286	1296	-0.0829	0.0259	0.3036
WW	HEI	HEA	-0.2141	1296	-0.2655	-0.1616	<.0001
WW	HI	GFP	-0.0059	1254	-0.0612	0.0495	0.8357
WW	HI	GN	0.4846	1253	0.4411	0.5259	<.0001
WW	HI	GW	0.0729	1253	0.0176	0.1277	0.0099
WW	HI	PGY	0.6407	1254	0.6069	0.6722	<.0001
WW	HI	HEA	-0.035	1254	-0.0902	0.0204	0.2152
WW	HI	HEI	-0.2163	1254	-0.2685	-0.1629	<.0001
WW	MAT	GFP	0.0259	1296	-0.0286	0.0802	0.3521
WW	MAT	GN	0.0451	1295	-0.0094	0.0993	0.1049
WW	MAT	GW	0.058	1295	0.0035	0.1121	0.0369
WW	MAT	PGY	0.0846	1296	0.0303	0.1384	0.0023
WW	MAT	HEA	0.7502	1296	0.7254	0.7731	<.0001
WW	MAT	HEI	-0.1361	1296	-0.1892	-0.0823	<.0001
WW	MAT	HI	-0.0456	1254	-0.1008	0.0097	0.1061
WW	TDM	GFP	-0.0385	1274	-0.0932	0.0165	0.1699
WW	TDM	GN	0.3762	1273	0.328	0.4224	<.0001
WW	TDM	GW	-0.093	1273	-0.1472	-0.0382	0.0009
WW	TDM	PGY	0.4036	1274	0.3566	0.4486	<.0001
WW	TDM	HEA	0.1491	1274	0.095	0.2024	<.0001
WW	TDM	HEI	0.2318	1274	0.1791	0.2831	<.0001
WW	TDM	HI	-0.4054	1254	-0.4506	-0.3581	<.0001
WW	TDM	MAT	0.1552	1274	0.1012	0.2084	<.0001
WW	VDW	GFP	-0.0353	1242	-0.0907	0.0204	0.2141
WW	VDW	GN	0.0846	1241	0.0291	0.1396	0.0029
WW	VDW	GW	-0.1109	1241	-0.1656	-0.0556	<.0001
WW	VDW	PGY	0.0489	1242	-0.0067	0.1042	0.085
WW	VDW	HEA	0.1313	1242	0.0762	0.1855	<.0001
WW	VDW	HEI	0.2557	1242	0.203	0.307	<.0001
WW	VDW	HI	-0.7027	1242	-0.7298	-0.6733	<.0001
WW	VDW	MAT	0.1363	1242	0.0813	0.1905	<.0001
WW	VDW	TDM	0.9258	1242	0.9174	0.9334	<.0001

Authors: Lianne Merchuk-Ovnat*, Roi Silberman*, Efrat Laiba, Andreas Maurer, Klaus Pillen, Adi Faigenboim, Eyal Fridman

Table S4 GWAS results for trait per se. The effect correspond to the percent difference between mean phenotypic value of homozygous for the wild allele comapre to carriers of the cultivated Barke allele within the whole HEB-25 population

Marker	Env	Trait	chr	CM	lod	effect
SCRI_RS_197263	WL	MAT	1H	88.9	5.74E+00	1.7
BOPA1_4962_1295	WL	VDW	1H	100	5.45E+00	-8.7
SCRI_RS_216088	WL	GFP	1H	122.2	7.62E+00	7.8
SCRI_RS_153896	WL	HEA	1H	132.4	9.75E+00	-3.6
BOPA2_12_30871	WL	MAT	2H	20	1.66E+01	2.7
BK_14	WL	GN	2H	20	7.87E+00	-19.7
BK_15	WL	GFP	2H	20	1.55E+01	-10.12
BK_15	WL	HEA	2H	20	3.69E+01	6.77
SCRI_RS_155067	WL	TDM	2H	57.4	6.19E+00	-6.8
SCRI_RS_196026	WL	GFP	2H	57.4	4.09E+01	17.6
SCRI_RS_196026	WL	VDW	2H	57.4	1.00E+01	-10
SCRI_RS_235063	WL	HEA	2H	57.4	5.78E+01	-7.7
SCRI_RS_12492	WL	HI	2H	57.4	5.29E+00	26.58
BOPA1_ABC08774_1_1_752	WL	MAT	2H	57.4	1.44E+01	-2.12
SCRI_RS_199987	WL	TDM	3H	40.7	2.57E+01	17.4
SCRI_RS_199987	WL	VDW	3H	40.7	2.45E+01	20
SCRI_RS_151711	WL	MAT	3H	103.8	6.17E+00	-2.1
SCRI_RS_120973	WL	GFP	3H	108	9.88E+00	9.06
SCRI_RS_120973	WL	HEA	3H	108	1.83E+01	-4.5
SCRI_RS_103215	WL	HEI	3H	109.2	9.89E+01	21
SCRI_RS_103215	WL	TDM	3H	109.2	6.60E+00	6.9
SCRI_RS_103215	WL	VDW	3H	109.2	8.36E+00	9.8
SCRI_RS_194316	WL	GFP	4H	51.4	7.72E+00	19
SCRI_RS_13766	WL	MAT	5H	106	2.00E+01	2.9
SCRI_RS_239128	WL	HEA	5H	115.3	2.97E+01	5.58
SCRI_RS_189371	WL	GFP	5H	118	8.07E+00	-6.7
SCRI_RS_138461	WL	GFP	7H	29.8	6.88E+00	-7.1
BOPA2_12_30894	WL	HEA	7H	34.2	2.18E+01	5.7
BOPA2_12_30894	WL	MAT	7H	34.2	1.46E+01	2.8
BOPA1_5772_1176	WW	GN	1H	72.6	7.37E+00	-12
BOPA1_5772_1176	WW	PGY	1H	72.6	4.75E+00	-8
BOPA2_12_31319	WW	GW	1H	92.4	7.29E+00	8.6

BOPA1_5336_400	WW	VDW	1H	100	4.68E+00	-7.5
SCRI_RS_106754	WW	TDM	1H	100	6.14E+00	-6.5
BOPA1_ABC05061_1_1_159	WW	HEA	1H	128	8.58E+00	-3.4
SCRI_RS_153896	WW	MAT	1H	132.4	8.16E+00	-1.8
BOPA1_3692_940	WW	GW	2H	2	7.67E+00	16.3
BK_15	WW	GFP	2H	20	8.64E+00	-5.16
BK_15	WW	HEA	2H	20	3.87E+01	6.71
BK_15	WW	MAT	2H	20	3.16E+01	3.6
SCRI_RS_167882	WW	TDM	2H	57.4	2.12E+01	-11.6
SCRI_RS_222769	WW	HI	2H	57.4	7.79E+00	6.14
SCRI_RS_222769	WW	MAT	2H	57.4	2.29E+01	-2.6
SCRI_RS_222769	WW	VDW	2H	57.4	1.94E+01	-12.9
SCRI_RS_235063	WW	GFP	2H	57.4	2.91E+01	11.5
SCRI_RS_235063	WW	HEA	2H	57.4	5.38E+01	-7.3
SCRI_RS_154973	WW	PGY	3H	40.7	5.37E+00	-9.2
BOPA2_12_31475	WW	HI	3H	44.8	3.19E+01	-18.9
SCRI_RS_196189	WW	TDM	3H	51.6	1.89E+01	11
SCRI_RS_196189	WW	VDW	3H	51.6	2.91E+01	19.4
SCRI_RS_171144	WW	TDM	3H	105	6.61E+00	10.54
BOPA1_ABC13753_1_2_167	WW	GN	3H	105	7.14E+00	-10.01
BOPA1_6069_304	WW	PGY	3H	108	1.13E+01	-10.5
SCRI_RS_138723	WW	MAT	3H	108	1.17E+01	-2
SCRI_RS_120973	WW	HEA	3H	108	2.10E+01	-4.8
SCRI_RS_103215	WW	GFP	3H	109.2	1.09E+01	7.2
SCRI_RS_103215	WW	HEI	3H	109.2	1.26E+02	21
SCRI_RS_103215	WW	HI	3H	109.2	2.63E+01	-15.2
SCRI_RS_103215	WW	VDW	3H	109.2	1.22E+01	12.3
BOPA1_3127_273	WW	HI	4H	54.8	4.93E+00	6.18
BOPA2_12_31385	WW	GN	4H	60	6.02E+00	11.9
BOPA1_41_695	WW	TDM	4H	100.6	5.67E+00	6.66
BOPA1_4771_380	WW	GW	5H	95	7.20E+00	-3.5
SCRI_RS_236583	WW	HEA	5H	115.3	3.01E+01	5.3
SCRI_RS_232705	WW	GFP	5H	118	1.05E+01	-5.9
BOPA1_272_944	WW	MAT	5H	118	2.03E+01	2.73
BOPA2_12_30894	WW	HEA	7H	34.2	2.92E+01	6.2
BOPA2_12_30894	WW	MAT	7H	34.2	1.83E+01	3
BOPA2_12_30083	WW	GFP	7H	45	9.89E+00	-7.2

Supplementary data
Manuscript Title: Genome scan identifies flowering-independent effects of barley HsDry2.2 locus on yield traits under water deficit
Authors: Lianne Merchuk-Ovnat*, Roi Silberman*, Efrat Laiba, Andreas Maurer, Klaus Pillen, Adi Faigenboim, Eyal Fridman

Table S5 QxE loci for the different traits over two years trials. The effect is calculated as the difference between the effect of the wild allele under WW and WL. Positive values indicate higher increasing or less reducing effect of the wild allele under WL. DR, Detection rate calculated across 200 repeated subsamples of 70% from HRB-25 population (see Methods)

Marker	Trait	chr	cM	DR[%]	effect
BOPA2_12_30304	PGY	1H	57.3	64	8.01
SCRI_RS_216088	PGY	1H	122.2	80	10.95
SCRI_RS_216088	GN	1H	122.2	91	15.37
SCRI_RS_188893	GFP	2H	20	63.5	-6.06
SCRI_RS_157207	VDW	2H	57.4	58.5	5.45
SCRI_RS_100054	PGY	2H	57.4	100	13.65
SCRI_RS_208320	GN	2H	57.4	95	19.08
SCRI_RS_196026	GFP	2H	57.4	78.5	6.2
SCRI_RS_185710	TDM	2H	57.4	100	4.69
SCRI_RS_146425	PGY	3H	44.8	100	13.24
SCRI_RS_230096	GN	3H	44.8	97	11.59
SCRI_RS_223894	VDW	3H	51.6	96.5	-8
SCRI_RS_173348	PGY	3H	51.6	86	7.7
SCRI_RS_173348	GN	3H	51.6	61.5	8.6
SCRI_RS_206510	PGY	3H	103.8	100	4.75
SCRI_RS_206510	GN	3H	103.8	54.5	6.76
SCRI_RS_138221	GN	5H	98.1	82.5	-13.47
SCRI_RS_231239	TDM	5H	109.7	73	-4.49
SCRI_RS_207174	GFP	6H	54.9	33.5	2.96
SCRI_RS_204148	PGY	6H	61	89.5	2.58
SCRI_RS_219709	TDM	7H	24.5	67	-4.65
SCRI_RS_187827	TDM	7H	43.8	68.5	-3.7

Supplementary data
Manuscript Title: Genome scan identifies flowering-independent effects of barley HsDry2.2 locus on yield traits under water deficit
Authors: Lianne Merchuk-Ovnat*, Roi Silberman*, Efrat Laiba, Andreas Maurer, Klaus Pillen, Adi Faigenboim, Eyal Fridman

Table S6 Pot experiment ANOVA

Analysis of variance (ANOVA) for the measured traits: total DM (TDM, gr/plant), plant grain yield (PGY, gr/plant), harvest index (HI), spike per plant, grain number of three first spike (GN- first three), grain number (GN all), grain weight of three first spike (GW- first three), grain weight (GW, all), days from planting to booting (HEA, days), ripening period (RIP, days), Flag leaf sheath (cm) length, stem diameter (cm), -1 flag leaf blade width (cm), -1 flag leaf blade length.

Source	Irrigation Treatment (d.f.=2) F ratio	Genotype (d.f.=1) F ratio	Irrigation x Genotype (d.f.=2) F ratio	d.f. error
**TDM**	57.05 ***	0.08	0.21	49
**PGY**	14.94 ***	8.83 **	2.82 ^	45
**HI**	10.58 **	7.76 **	0.53	44
**Spike number**	4.27 *	2.1	0.4	48
**GN- three first spike**	1.01	4.72 *	2.8 ^	38
**GN- all**	1.78	3.09 ^	2.54 ^	40
**TGW- three first spike**	3.09 ^	3.67 ^	1.06	38
**TGW- all**	10.62 ***	3.48 ^	1.36	38
**HEA**	7.27 ***	6.43 *	0.45	49
**GFP**	4.03 *	4.43 *	0.21	42
**FL sheath length**	2.11	4.98 *	1.36	42
**Stem diameter**	1.07	8.77 ***	0.05	48
**minus 1 FL width**	5.21 **	9.83 **	2.11	42
**minus 1 FL length**	5.34 ***	26.22 ***	0.79	42
**Senescence**	10.73 ***	4.17 *	0.33	47

*, **, *** represent $P < 0.05$, 0.01 and 0.001, respectively.

^ represent $P < 0.1$

Supplementary data

Manuscript Title: Genome scan identifies flowering-independent effects of barley HsDry2.2 locus on yield traits under water deficit **Authors:** Lianne Merchuk-Ovnat*, Roi Silberman*, Efrat Laiba, Andreas Maurer, Klaus Pillen, Adi Faigenboim, Eyal Fridman

File S1 R-script for the GWAF analysis

```
library(GWAF)

library(kinship2)


ped=read.csv(file="ped_file.csv", header=TRUE,sep=",")

kmat<-makekinship(ped$famid,ped$id,ped$fa,ped$mo)

kmat<-kmat*2

save(kmat,file="heb_interaction_kinship.Rdata")


lmepack.int.batch(phenfile="trait.csv",genfile="geno_file.csv",
pedfile="ped_file.csv",kinmat="heb_interaction_kinship.Rdata",phen="trait",
outfile="trait.lme.quant.int.csv",covars=c('dry'), cov.int='dry', sub='N',
sep.ped=',', sep.phe=',', sep.gen=',',col.names=F)
```
