# Supplementary material for: Genome scan identifies flowering-independent effects of barley HsDry2.2 locus on yield traits under water deficit
Source: J Exp Bot. 2018 Jan 8;69(7):1765–79. doi: 10.1093/jxb/ery016 (PMC5888960; doi:10.1093/jxb/ery016)
Supplement: Supplementary figure legends [file ery016_suppl_supplementary_figure_legends.docx]

**Supplementary figure legends:**

**Fig. S1** (A) High resolution melting analysis for the peak marker BOPA2_12_30265 for the genotyping of BC_2_F_2_ (self of backcrossing line HEB-04-96) segregating population in the HsDry2.2 locus. (B) Sanger sequencing showing the different alleles for the BOPA2_12_30265 SNP.

**Fig. S2** The effects of water limitation on control plants grown in a semi-controlled, high content plant phenotyping (HCPP) set-up. (A) The experimental set up for the 2015 and 2016 experiments included the HEB-25 lines and control plants. Plants were grown in pairs of troughs, under well-watered (WW) or water-limited (WL) conditions. (B) Accumulated irrigation and (C) stomatal conductance as a function of days from sowing, under the WW (blue) or WL (red) conditions, during 2016. (D) A panoramic view over the pot experiment at ripening stage. (E) Field capacity (%) as a function of days after planting (DAP) for the different genotypes. Seedlings were planted into small pots (60g soil and 300g water, 100% field capacity), at 28 DAP plants were transplanted into medium pots (190g soil and 1170g water), ant at 52 DAP plants were into large pots (460g soil and 2500g water). The Early WL initiated at planting and the Late WL at 52 DAP, about two weeks before booting. Pots were weighed manually before and after irrigation, keeping the well-watered (WW) pots between 60-90% of field capacity and the water limited pots (WL) at 40-60%. (F) Stomatal conductance measured for cv. Barke control at 56 and 69 DAP under the different irrigation treatments. Arrows in figure 2B and 2C mark the mean heading date of the whole HEB-25 population.

**Fig. S3** Reaction norm of *HsDry2.2* is illustrating the mean values of vegetative dry matter (VDW) under WW and WL conditions, in the whole HEB-25 population. The three genotypic groups of plants homozygous for the Barke cultivated allele (Hv/Hv), homozygous for the wild allele (Hs/Hs) and heterozygous (Hv/Hs) are depicted by blue, red and gray lines, respectively.

**Fig. S4** Reaction norms of *HsDry2.2* are illustrating the mean values of plant grain number (GN) under WW and WL (reaction norms) conditions, in the a) HEB-16 and b) HEB-05 families. Homozygous for the Barke cultivated allele (Hv/Hv) and for the wild allele (Hs/Hs) are depicted by blue and gray lines, respectively. Heterozygous plants were excluded from analysis due to low number of replicates (<6).

**Fig. S5** (A) Representative photos for canopy structure modifications, showing the carriers of the wild allele (Hs/_) have on average longer sheath, and narrower-shorter leaf blades (of FL and -1FL). (B) Stem cuts after harvest at the base of the spike (Late WL) showing Hs/_ plants to have reduced stem diameter, which also appear to be thicker than in the cultivated allele.

**Fig. S6** Air minimum and maximum daily temperatures monitored over the pot experiment season.

**Fig. S7**Sangersequencing results for the *HvCEN* gene identifies segregation for the P135A at the HEB-04 family. Line HEB-04-02
